# Plasmacytoid and CD141+ Myeloid Dendritic Cells Cooperation with CD8+ T Cells in Lymph Nodes is Associated with HIV Control

**DOI:** 10.1002/mco2.70354

**Published:** 2025-09-12

**Authors:** Joana Vitallé, Sara Bachiller, Beatriz Dominguez‐Molina, Eirini Moysi, Sara Ferrando‐Martínez, María Inés Camacho‐Sojo, Isabel Gallego, Alberto Pérez‐Gómez, María Reyes Jiménez‐Leon, Carmen Gasca‐Capote, Francisco José Ostos, Mohammed Rafii‐El‐Idrissi Benhnia, Laura E. Via, Antonio Mochón, Luis Fernando López‐Cortes, Constantinos Petrovas, Richard A. Koup, Ezequiel Ruiz‐Mateos

**Affiliations:** ^1^ Institute of Biomedicine of Seville, IBiS/Virgen Del Rocío University Hospital/CSIC/University of Seville Clinical Unit of Infectious Diseases, Microbiology and Parasitology Seville Spain; ^2^ Department of Medical Biochemistry Molecular Biology, and Immunology School of Medicine University of Seville Seville Spain; ^3^ Tissue Analysis Core Immunology Laboratory Vaccine Research Center NIAID NIH Bethesda Maryland USA; ^4^ Tuberculosis Research Section Laboratory of Clinical Immunology and Microbiology, and Tuberculosis Imaging Program Division of Intramural Research NIAID NIH Bethesda Maryland USA; ^5^ Otorhinolaryngology Service Virgen Del Rocío University Hospital Seville Spain; ^6^ Department of Laboratory Medicine and Pathology University of Lausanne University Hospital of Lausanne (CHUV) Lausanne Switzerland

**Keywords:** HIV, dendritic cell (DC), plasmacytoid DC, CD141 myeloid DC, CD8 T cell, lymph node

## Abstract

Dendritic cells (DC) are known to modulate antiviral immune responses; however, the knowledge about the role of different DC subsets in antiviral T cell priming in human tissues remains uncompleted. In the context of HIV infection, we determined the phenotype and location of plasmacytoid and CD141+ myeloid DCs (pDCs and mDCs) in lymph nodes of people living with HIV (PLWH). We found an interaction between pDCs and CD141+ mDCs with CD8+ T cells, being associated with participants’ viral levels in blood and tissue. Moreover, we demonstrated a higher and more polyfunctional superantigen‐ and HIV‐specific CD8+ T cell response after the coculture with Toll‐like receptor (TLR)‐primed pDCs and CD141+ mDCs. Last, we showed the potential of programmed cell death‐1 (PD‐1) blocking using pembrolizumab to further increase antigen‐specific CD8+ T cell response along with TLR agonists. Therefore, these results showed a cooperation between pDCs, CD141+ mDCs and CD8+ T cells in lymph nodes of PLWH, which is associated with higher HIV control, highlighting the importance of DC subsets crosstalk to achieve a more potent anti‐HIV response and support the use of DC‐based immunotherapies for HIV control.

## Graphical abstract

Plasmacytoid and CD141+ myeloid dendritic cells cooperate with CD8+ T cells in lymph nodes, which is associated with HIV levels in blood and tissue. The interaction with primed dendritic cells with Toll‐like receptor agonists increase HIV‐specific CD8+ T cell response and PD‐1 blocking further enhance this response.

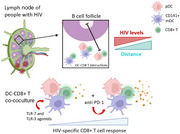

## 1 Introduction

Despite the efficacy of antiretroviral therapy (ART) to suppress HIV replication, it fails to eradicate the virus, due to the maintenance of anatomical and cellular HIV reservoirs in people living with HIV (PLWH) [1, 2]. Therefore, the main goal of current immunotherapeutic strategies is HIV eradication or permanent viral remission off ART. Among all the new strategies to achieve the permanent viral remission, dendritic cell (DC)‐based immunotherapies have shown very promising results in the last years, thanks to their ability to modulate virus‐specific immune responses [3, 4, 5, 6, 7].

The DCs regulate the immune response through different pathways depending on the DC subpopulation. Myeloid DCs (mDCs) carry out their function after the stimulation through different Toll‐like receptors (TLR) such as TLR‐3. They are divided in three subsets: (1) CD141+ or DC1 mDCs, with a principal role in antigen cross‐presentation and CD8+ T cell costimulation; (2) CD1c+ or DC2 mDCs, which modulate CD4+ T cell response; and (3) CD16+ mDCs, involved in inflammatory responses [8, 9]. Plasmacytoid DCs (pDCs) are important drivers of both innate and adaptive immune responses. They are known as the main producers of type‐I interferon (IFN) via TLR‐7/9 stimulation, which orchestrates the antiviral response by inducing IFN‐stimulated genes and apoptosis of infected cells [8, 9, 10].

Several studies have previously reported the relevance of both pDCs and CD141+ mDCs in the anti‐HIV response. pDCs were involved in HIV spontaneous control, reducing HIV replication in elite HIV controllers [11]. In addition, TLR‐7 stimulation increased pDC activation and the expression of IFN‐related genes, which in turn induced an HIV‐specific T cell response. This phenomenon was previously described in PLWH, both in vitro [6, 7] and in vivo [3, 4]. Although little is known about the role of CD141+ mDCs in HIV control, priming mDCs with TLR‐3 and STING agonists has been shown to induce polyfunctional HIV‐specific CD8+ T cell responses in vitro and in vivo using a humanized BLT mouse model [12]. Furthermore, the expansion of XCR1+ mDCs, the murine counterpart of human CD141+ mDCs, improved viral control in chronic lymphocytic choriomeningitis virus (LCMV) mouse model [13]. All these findings showed the important role of pDCs, and probably CD141+ mDCs, separately, in the induction of HIV‐specific CD8+ T cell response. However, if both DC subsets can interact at the same time with CD8+ T cells increasing their antiviral response in humans is still unknown. Moreover, thanks to the capacity of TLR agonists to stimulate DCs, among others, and previous clinical trials showing the ability of TLR agonists to improve antiviral response and as latency reversal agents in the context of HIV/SIV infection [3, 4, 14, 15], highlighted their potential as immunotherapeutic strategy alone or in combination with other compounds for PLWH.

DC–T cell interactions occur in lymphoid tissues. Lymph nodes (LNs) are highly structured secondary lymphoid organs, where cell–cell interactions modulate adaptive immune responses [16]. It is also known that LNs are targeted by HIV infection, inducing the migration and redistribution of several immune cells. Among others, follicular CD8+ T cells (fCD8) accumulate during HIV infection, which have been associated with viral control [17, 18, 19]. Regarding DCs, various works studied the frequency and location of total or specific DCs subsets in LNs in mice [12, 20, 21]. In humans, Moysi et al. [22] analyzed pDCs and CD141+ mDCs in tonsils and follicular DCs in LNs from PLWH, showing the proportion and location of these cell types in lymphoid tissues. Of note, Brewitz et al. [23] showed that CD8+ T cells were able to recruit both pDCs and XCR1+ mDCs to LNs, and this cooperation increased antigen‐specific CD8+ T cell fraction and proliferation in mice infected with modified vaccinia virus Ankara. However, in the context of viral infection in humans, including HIV infection, the knowledge of the role of different DC subsets in T cell priming in lymphoid tissues is incomplete.

In this work, we characterized the proportion, phenotype and location of pDCs and CD141+ mDCs, and their interaction with CD8+ T cells, in LNs of PLWH, in relation to HIV levels. We also determined the capacity of pDCs and CD141+ mDCs to increase antigen‐specific CD8+ T cell response, by in vitro coculture experiments with primed DCs with Poly I:C (TLR‐3 agonist) and GS‐9620 (TLR‐7 agonist). Last, we investigated the potential of PD‐1 blockade along with TLR stimulation to boost antigen‐specific CD8+ T cell response.

## 2 Results

### 2.1 Association of pDCs and CD141+ mDCs with Viral Load in Peripheral Blood and LNs from PLWH

**FIGURE 1:**
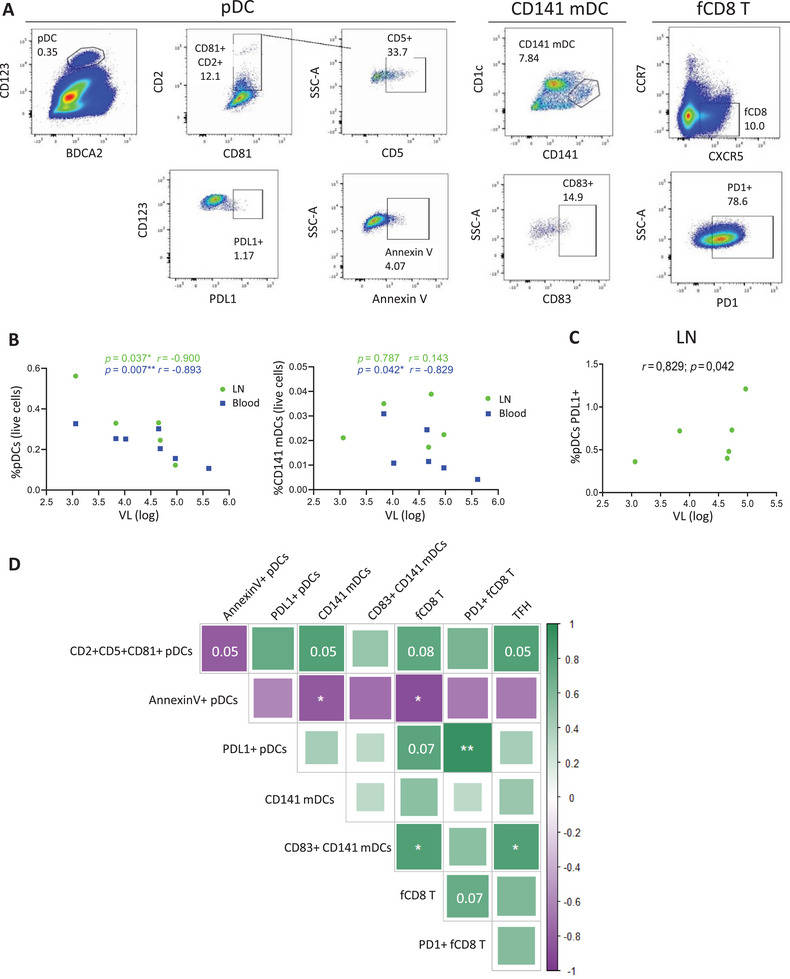
pDCs and CD141+ mDCs are associated with viral load and follicular T cells. (A) Representative pseudocolor dot plots showing the gating strategy for pDC, CD141+ mDC, and fCD8+ T cell subsets and markers. (B) Correlations between pDC and CD141+ mDC percentages with viral load in lymph nodes (LN, green) and peripheral blood (blood, blue). (C) Correlation of PD‐L1+ pDC percentage with viral load in LN (green). (D) Correlation matrix representing the associations between pDCs, CD141+ mDCs, and follicular CD8+ (fCD8) and CD4+ (TFH) T cells in LN. Green and purple colors represent positive and negative correlations, respectively. The intensity of the color and the size of the squares indicate the *R* coefficient. Spearman test was used (*n* = 7) and ROUT method was utilized to identify and discard outliers (*Q* = 0.1%). *p < 0.05, **p < 0.01. *p* Values between 0.05 and 0.1 were considered as tendency and were shown as numbers.

CD141+ mDCs and pDCs have been previously related to HIV control due to their capacity to induce HIV‐specific cellular responses [6, 11, 12]. Hence, to investigate the potential role of these cells in HIV control, not only in blood but also in lymphoid tissue, we first analyzed these DC subsets and T cells in peripheral blood and LNs of ART naïve PLWH by ex vivo flow cytometry (*n* = 8) (Figures 1A and S1A,B and Table S1) and their association with participants’ viral load. As occurs in non‐HIV population [10], in both blood and LNs of PLWH, the percentage of pDCs was very low (median [interquartile range (IQR)] (% within live cells); LN: 0.33 [0.215–0.774] and blood: 0.25 [0.155–0.302]) comparing with other cell types as CD4+ or CD8+ T cells (median [IQR] (% within live cells); CD4+ T LN: 42.5 [40.1–47.8], CD4+ T blood: 38.8 [31.3–41.4], CD8+ T LN: 24.1 [21.2–26.3] and CD8+ T blood: 35.2 [30.5–38.5]), and the percentage of CD141+ mDCs was even lower (median [IQR] (% within live cells); LN: 0.023 [0.020–0.035] and blood: 0.011 [0.009–0.024]) (Figure S1A–C). Percentages of fCD8 and follicular CD4+ T cells (TFH) in LN are shown in Figure S1D. Negative correlations between viral load and the percentage of pDCs in both blood or LN were found (Figure 1B, left panel); however, in CD141+ mDCs, this negative correlation was only observed in peripheral blood (Figure 1B, right panel). Furthermore, the expression of the integrin CD103 and TLR‐9 on pDCs from blood was positively associated with viral load (Figure S2A). The expression of PD‐L1 on pDCs in LN, the ligand of the immune checkpoint inhibitor PD‐1, which reflects pDC activation, was positively correlated to viral load (Figure 1C). These data suggested a potential role of CD141+ mDCs and pDCs in HIV control.

### 2.2 pDCs and CD141+ mDCs are Associated with fCD8 T Cells in LNs of PLWH

In addition to the associations with viral load, we also found that pDCs and CD141+ mDCs were related to each other and to follicular T cells in LN of PLWH. First, we observed the presence of specific pDC and CD141+ mDC subsets in LNs of PLWH: CD2+ CD5+ CD81+ pDCs, a subset that has been described as a strong inducer of T cell proliferation [24], and CD83+ CD141+ mDCs, being CD83 a costimulatory molecule that reflects DC maturation with a key role in the induction of T cell proliferation [25] (Figure 1A). CD2+ CD5+ CD81+ pDCs tended to be negatively associated with apoptotic pDCs (annexin V+) and positively associated with total CD141+ mDCs, fCD8, and TFH in LNs of PLWH (Figure 1D). The percentage of apoptotic pDCs was negatively associated with the frequency of CD141+ mDCs and fCD8. As expected, PD‐L1+ pDCs were positively correlated with fCD8 expressing PD‐1. Moreover, CD83+ CD141+ mDCs were also positively associated with fCD8 and TFH (Figure 1D). Last, CD141+ mDCs expressing markers of activation/maturation (CD86, CD40, and PD‐L1) and homing to LN (CCR7) were positively correlated to pDCs that migrate to LN (CCR7+) (Figure S2B,C) and inversely associated with exhausted pDCs (Tim‐3+) (Figure S2B,D). Overall, these results showed an association between pDCs, CD141+ mDCs, and CD8+ T cells in LN from PLWH, which may be relevant to HIV control.

### 2.3 pDC, CD141+ mDC, and CD8+ T Cell Interaction in LN of PLWH

**FIGURE 2:**
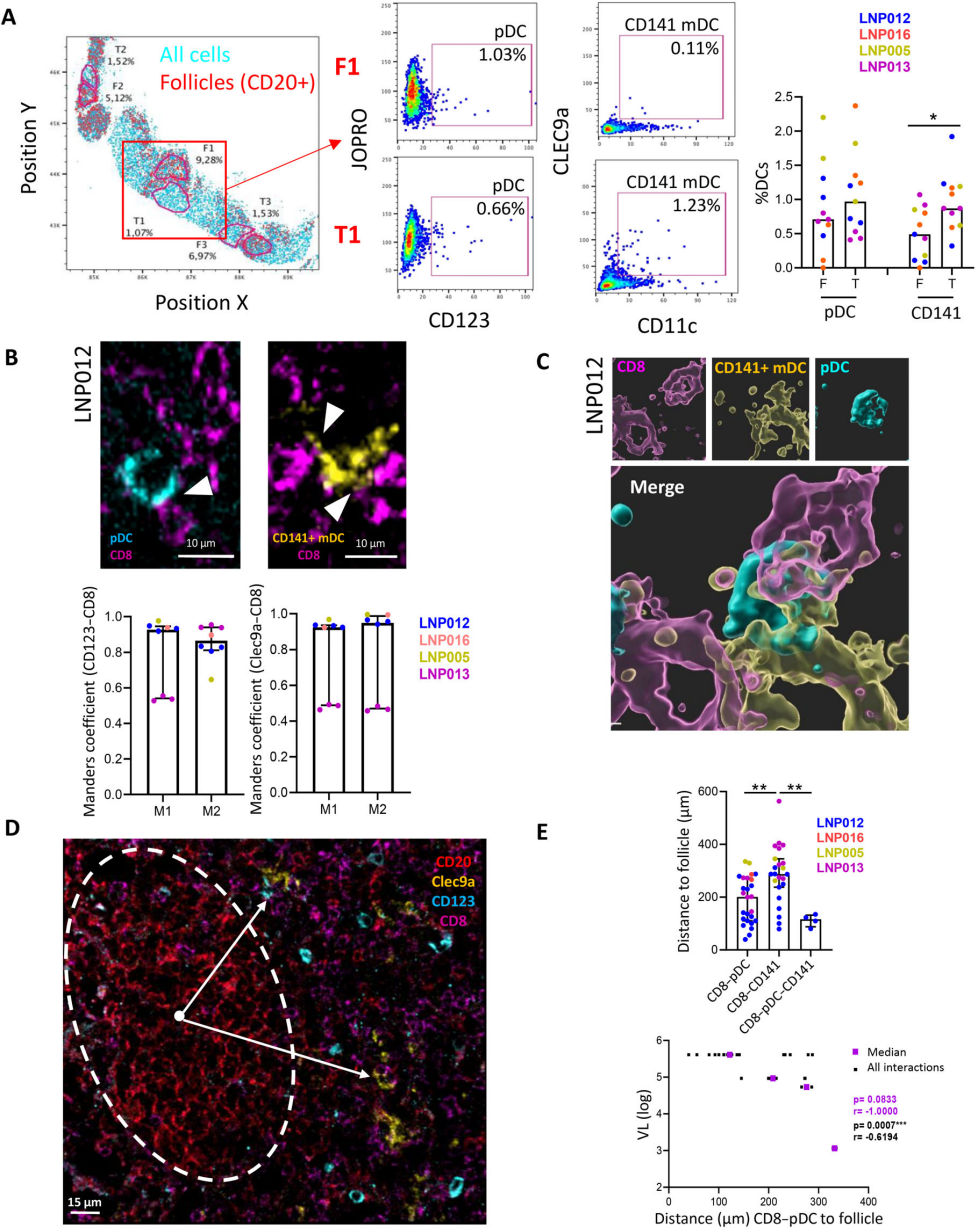
pDC, CD141+ mDC, and CD8+ T cell interaction in LN of PLWH. (A) Dot plot graphs showing representative data of histocytometry analysis of a LN (LNP012), all cells are represented in blue and CD20+ cells (follicles) in red; follicles (F) and the closest T cell zones (T) were gated and pDC and CD141+ mDC percentages were determined (left). Bar graphs showing the percentages of pDCs and CD141+ mDCs of LNs from PLWH within the follicles (F) and T cell zones (T); each dot represents a follicle (right). (B) Representative confocal images showing the contact (white arrowheads) between pDCs (cyan) with CD8+ T cells (magenta) and CD141+ mDCs (yellow) with CD8+ T cells (magenta) (up panels). M1 and M2 Manders coefficients of CLEC9a (CD141)–CD8 and CD123 (pDC)–CD8 according to the colocalization patterns in confocal images (bottom panels). (C) Representative 3D microscopy image showing the contact between CD8+ T cells (magenta), CD141+ mDCs (yellow), and pDCs (cyan) in LN from PLWH (LNP012). (D) Representative images showing the analysis of the distance of DC–CD8+ T cell interaction to the follicles in LN; CD8+ T cells (magenta), CD141+ mDCs (yellow), pDCs (cyan), and follicles (red). (E) Bar graphs showing the distance (µm) of pDC–CD8+ T cell and CD141+ mDC–CD8+ T cell interaction and triple interaction to the follicle (up panel). Each dot represents an interaction. Correlation of the distance of pDC–CD8+ T cell interaction to the follicle with viral load (VL). All interactions are shown in black and the median of the interactions in each participant are represented in purple (bottom panel). PLWH are represented with different colors. Wilcoxon and Spearman tests were used (*n* = 4). *p < 0.05, **p < 0.01, ***p < 0.001.

The next step was to determine the location and interaction of pDCs, CD141+ mDCs, and CD8+ T cells in the tissue by confocal microscopy in four LNs from ART naïve PLWH (Table S1). First, DC distribution in LN was determined by histocytometry, which allows the analysis of the location of complex immune‐phenotypes across the tissue microanatomy in a quantitative and automatic manner, based on image acquisition by confocal microscopy [26]. Representative follicles (CD20+) and the closest T cell zones to the follicles were selected in LNs (Figure 2A). pDCs were found both inside and outside the follicles, while CD141+ mDCs were mainly located outside the follicles (Figure 2A). Additionally, microscopy images showed close contacts between pDCs or CD141+ mDCs with CD8+ T cells in all the studied LNs (Figure 2B up panels and Figure S3A, white arrows). Furthermore, the Manders coefficient revealed that the colocalization of CLEC9a/CD8 and CD123/CD8 was closed to 1.0 in most of the LNs analyzed (Figure 2B, bottom panels). Importantly, contacts between the three cell populations were only observed in one of the four studied participants, specifically the one showing the highest viral load (LNP012, viral load: 5.61 Log HIV‐RNA copies/mL) (Figure 2C). We also determined the distance of pDCs and CD141+ mDCs along with CD8+ T cells to the center of the follicle (Figure 2D). In accordance with the results obtained by histocytometry, the interaction between pDCs and CD8+ T cells, as well as the triple interaction, occurred closer to the follicles than CD141+ mDC–CD8+ T cell interaction (Figure 2E, up panel). Of note, the distance of pDC–CD8+ T cell interaction to the follicle was inversely correlated to viral load (Figure 2E, bottom panel), indicating that pDCs and CD8+ T cells were interacting closer to the follicle in the participants that displayed a higher HIV viremia. Last, to study the level of HIV infection in tissue and the association with DC–CD8+ T cell interaction, the capsid protein p24 was measured in three of the LNs (Figure S3B). The analyzed LNs showed a different percentage of HIV‐infected cells (Figure S3C, left), which correlated with plasma viral load (Figure S3C, right). Furthermore, in the same way as viremia, the percentage of p24+ cells was inversely associated with the distance of pDC–CD8+ T cell interaction to the follicles (Figure S3D). Therefore, our results demonstrated an interaction between pDCs, CD141+ mDCs, and CD8+ T cells in LN from PLWH and showed that the distance of pDC–CD8+ T cell interaction to the follicle was related to viral levels in blood and tissue.

### 2.4 The Interaction of Primed pDCs and CD141+ mDCs with CD8+ T Cells Induces a Higher Antigen‐Specific CD8+ T Cell Response in HIV− Donors and PLWH

**FIGURE 3:**
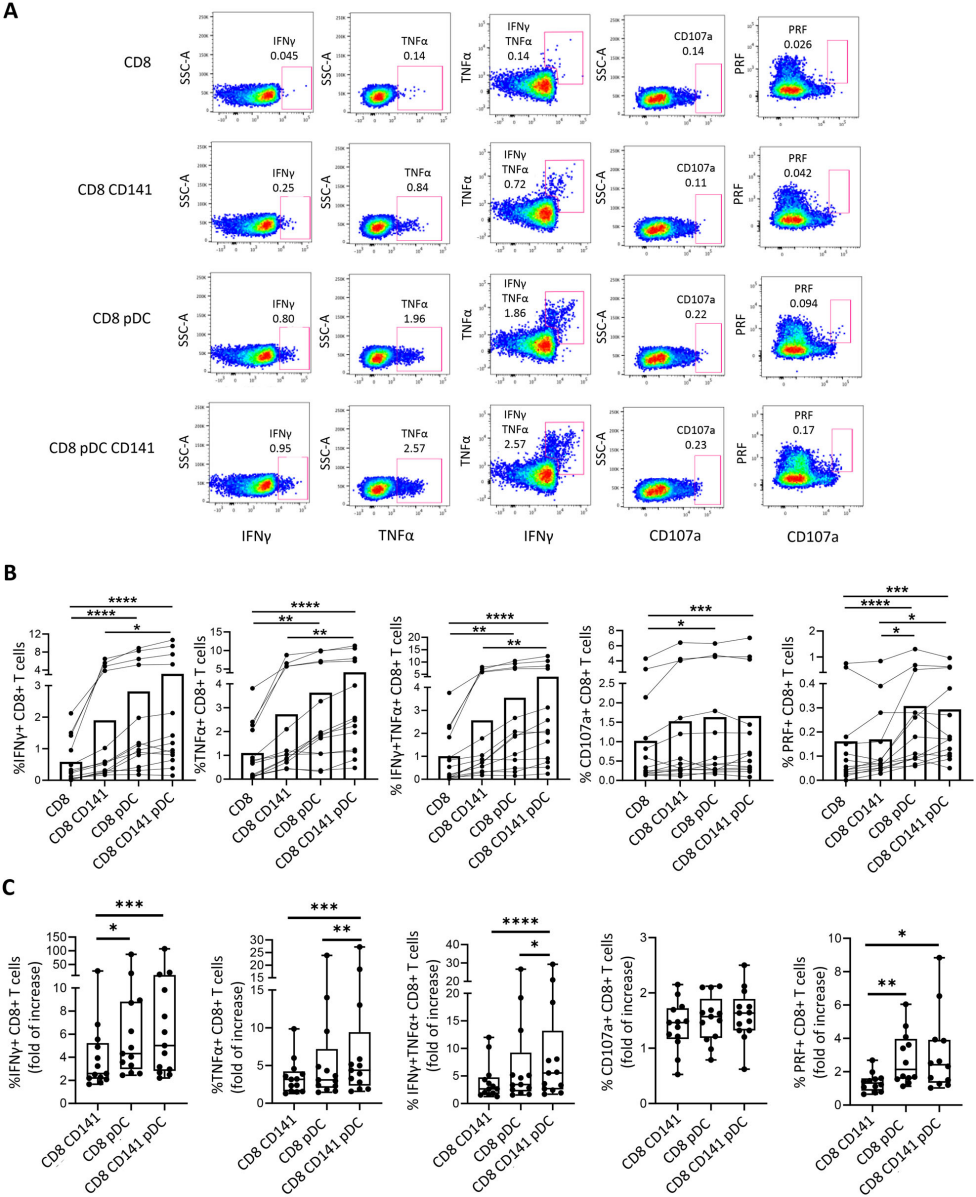
The interaction of primed pDCs and CD141+ mDCs with CD8+ T cells induces a higher SEB‐specific CD8+ T cell response in HD. (A) Representative pseudocolor dot plots and (B) bar graphs showing the percentages of IFNγ+, TNFα+, IFNγ+TNFα+, CD107+, and perforin (PRF)+ CD8+ T cells after prestimulated DC–CD8+ T cell coculture in the presence of enterotoxin type B (SEB) in HD. (C) Bar graphs showing the fold of increase of SEB‐specific IFNγ+, TNFα+, IFNγ+TNFα+, CD107+, and PRF+ CD8+ T cells after DC coculture, normalizing data with only CD8+ T cell condition. Each dot represents a participant. Friedman test was used (*n* = 13) and ROUT method was utilized to identify and discard outliers (*Q* = 0.1%). *p < 0.05, **p < 0.01, ***p < 0.001, ****p < 0.0001.

To investigate the effect of the interaction of pDCs and CD141+ mDCs with CD8+ T cells in antiviral response, pDCs and CD141+ mDCs were in vitro prestimulated with TLR‐7 and TLR‐3 agonists, respectively, and cocultured with CD8+ T cells. Then, the antigen‐specific memory CD8+ T cell response was analyzed by flow cytometry (Figure S4A). This was performed in both HIV− donors (HD) (*n* = 13) and PLWH (*n* = 6); due to the difficulty to obtain large volume of fresh blood samples from ART naïve PLWH, two PLWH on ART with undetectable viral load were also included exclusively for these coculture experiments (Table S1). In HD, although participants showed a variable background activation (the condition including only CD8+ T cells), we observed that memory CD8+ T cells showed a higher enterotoxin type B (SEB)‐specific production of IFNγ and TNFα, along with a higher expression of the degranulation marker CD107a and cytotoxicity marker perforin (PRF), when cocultured with primed pDCs and CD141+ mDCs (Figure 3A,B). In general, the interaction CD141+ mDCs–CD8+ T cell did not show a significant increase of SEB‐specific CD8+ T cell response, while an induced response was observed when only pDCs were included (Figure 3A,B). In terms of cytokine production, the coculture with only CD141+ mDCs showed the lowest SEB‐specific CD8+ T cell response, while the highest response was observed after the coculture with both pDCs and CD141+ mDCs (Figure 3A,B). In fact, when we normalized the data with the condition of CD8+ T cells alone, we found that the fold of increase in cytokine production (IFNγ and TNFα) was higher after the coculture with both pDCs and CD141+ mDCs in comparison with only pDCs or only CD141+ mDCs (Figure 3C). In terms of the cytotoxic capacity (PRF) of CD8+ T cells, no differences were found between only pDC and both pDC and CD141+ mDCs, but again, a lower fold of increase was observed after the coculture with only CD141+ mDCs (Figure 3C). By comparing the cytokine production and cytotoxic capacity of SEB‐stimulated CD8+ T cells with the nonstimulated ones, we confirmed that DC‐mediated increase in CD8+ T cell response was mainly antigen specific (Figure S4B).

As TLR agonists are known to induce DC activation and, consequently, increase the antiviral adaptive immune response [3, 12], we compared the SEB‐specific CD8+ T cell response after DC–CD8 T cell coculture with and without previous DC priming using TLR agonists in four of the HD, to confirm the relevance of DC priming and the immunotherapeutic potential of TLR agonists. Although no significant differences were found probably due to the sample size, the cytokine production (IFNγ and TNFα) by CD8+ T cells was 1.48–2.07 fold higher after the cocultured with primed DCs, comparing with non‐prestimulated DCs (Figure S4C). In contrast, we did not find any increase regarding the cytotoxic capacity (CD107a and PRF) (Figure S4C). The level of TLR‐mediated increase in CD8+ T cell response was associated with a higher CD8+ T cell cytokine production after pDC and CD141 mDC coculture (Figure S4D), suggesting a key role of TLR‐mediated priming in inducing DC‐dependent antigen‐specific CD8+ T cell cytokine production.

**FIGURE 4:**
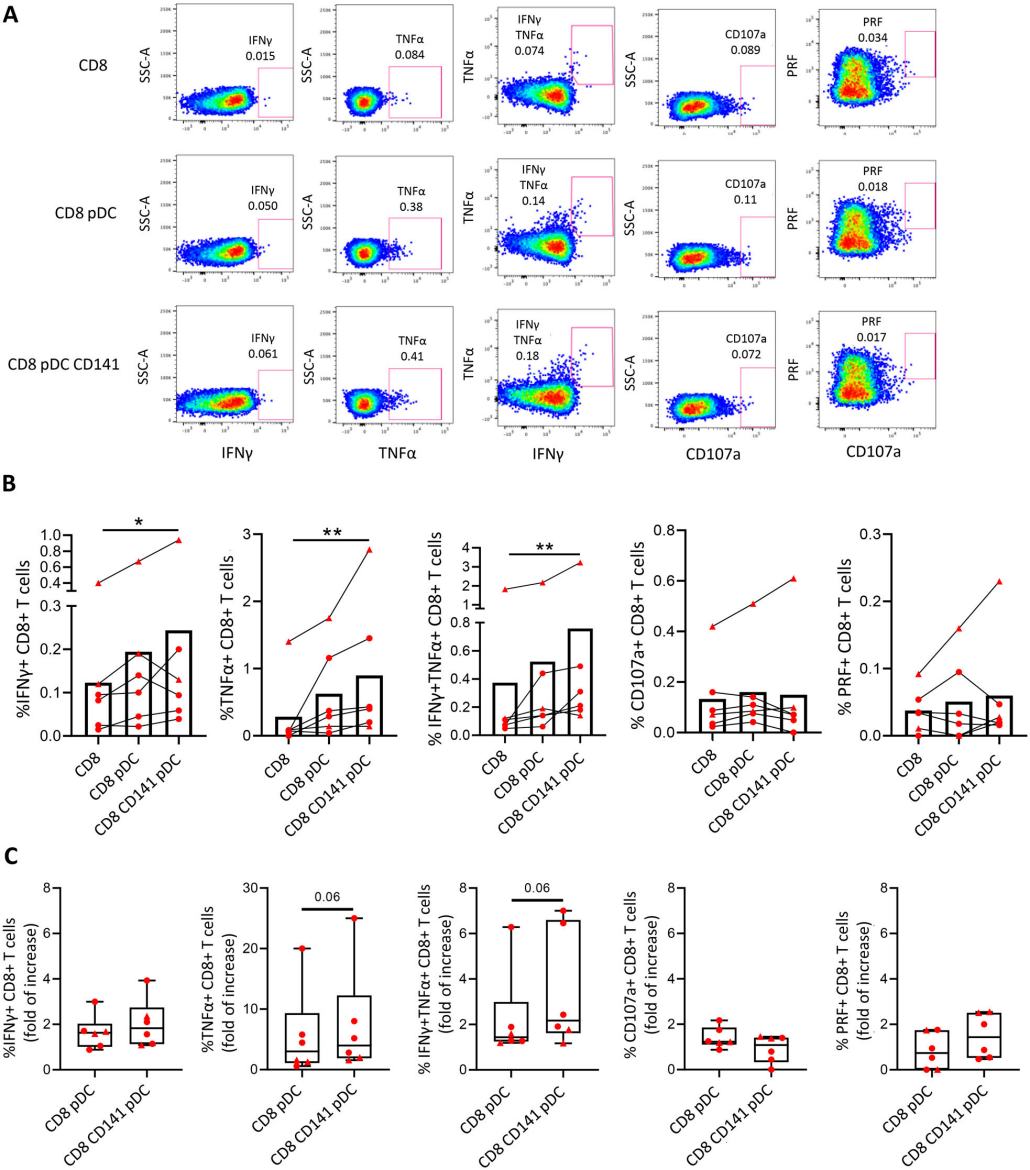
The interaction of primed pDCs and CD141+ mDCs with CD8+ T cells induces a higher HIV‐specific CD8+ T cell response in PLWH. (A) Representative pseudocolor dot plots and (B) bar graphs showing the percentages of IFNγ+, TNFα+, IFNγ+TNFα+, CD107+, and perforin (PRF)+ CD8+ T cells after prestimulated DC–CD8+ T cell coculture in the presence of HIV Gag peptides in PLWH. (C) Bar graphs showing the fold of increase of Gag‐specific IFNγ+, TNFα+, IFNγ+TNFα+, CD107+, and PRF+ CD8+ T cells after DC coculture, normalized with only CD8+ T cell condition. Red triangles represent PLWH under ART and red circles, naïve for treatment. Friedman and Wilcoxon tests were used (*n* = 6) and ROUT method was utilized to identify and discard outliers (*Q* = 0.1%). *p < 0.05, **p < 0.01, ***p < 0.001, ****p < 0.0001. *p* Values between 0.05 and 0.1 were considered as tendency and were shown as numbers.

Then, the same experiment was carried out with cells from PLWH. In accordance with the results of HD, when CD8+ T cells were cocultured with primed pDCs and CD141+ mDCs in presence of HIV Gag peptides, CD8+ T cells showed a higher HIV‐specific IFNγ and TNFα production after the interaction with both DC subsets. In this case, no significant differences were observed regarding degranulation or cytotoxic capacity (Figure 4A,B). When we normalized the data with the condition of CD8+ T cells alone, we found that the fold of increase in cytokine production (IFNγ and TNFα) tended to be higher after the coculture with both pDCs and CD141+ mDCs comparing with only pDCs (Figure 4C). We did not observe a different pattern on the participants on ART (triangles) and off ART (circles).

### 2.5 The Interaction of Primed pDCs and CD141+ mDCs with CD8+ T Cells Induces a More Polyfunctional Antigen‐Specific CD8+ T Cell Response

**FIGURE 5:**
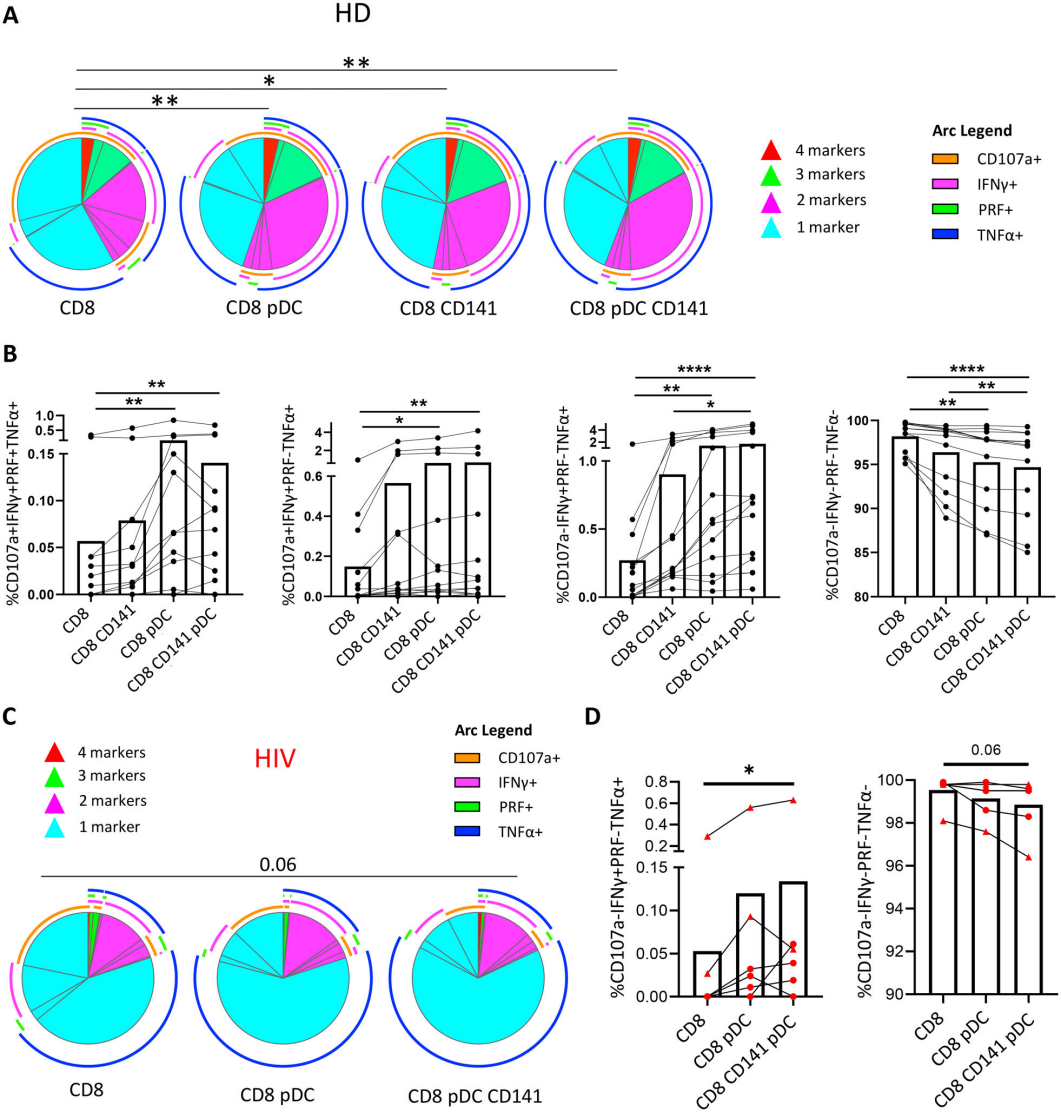
The interaction of primed pDCs and CD141+ mDCs with CD8+ T cells induces a more polyfunctional antigen‐specific CD8+ T cell response. (A) Pie charts representing SEB‐specific CD8+ T cell polyfunctionality after DC–CD8+ T cell coculture in HD. Each sector represents the proportion of CD8+ T cells expressing 4 (red), 3 (green), 2 (pink), and 1 (blue) function. Arcs represent the type of function (CD107a, IFNγ, PRF, and TNFα) expressed in each sector. (B) Bar graphs showing the percentage of CD8+ T cells expressing different combinations of studied functions in HD. (C) Pie charts representing HIV‐specific CD8+ T cell polyfunctionality and (D) bar graphs showing the percentage of CD8+ T cells expressing different combinations of studied functions after DC–CD8+ T cell coculture in PLWH. Each dot represents a participant; black circles represent HD, red circles ART naïve PLWH and red triangles PLWH on ART. Permutation and Friedman tests were used (HD, *n* = 13; PLWH, *n* = 6) and ROUT method was utilized to identify and discard outliers (*Q* = 0.1%). *p < 0.05, **p < 0.01, ****p < 0.0001. *p* Values between 0.05 and 0.1 were considered as tendency and were shown as numbers.

Afterward, we determined the quality of the CD8+ T cell response after DC coculture through a polyfunctionality analysis. Pie chart graphs showed a more polyfunctional SEB‐specific CD8+ T cell response when they were cocultured with either pDCs, CD141+ mDCs, or both, compared with CD8+ T cells alone (Figure 5A). Moreover, when we focused on the different combinations of the studied markers, we observed a higher percentage of CD8+ T cells expressing four markers (CD107a+ IFNγ+ PRF+ TNFα+), three markers (CD107a+ IFNγ+ PRF− TNFα+) or two markers (CD107a− IFNγ+ PRF− TNFα+) simultaneously, after the coculture with pDCs alone or with both pDCs and CD141+ mDCs (Figure 5B). In line with these results, CD8+ T cells cocultured with pDCs and both DC subsets also showed a lower percentage of negative cells for all the markers (Figure 5B). Regarding PLWH, the HIV‐specific response by CD8+ T cells tended to be more polyfunctional after the coculture with primed pDCs and CD141+ mDCs, in comparison with CD8+ T cells alone (Figure 5C). Furthermore, a higher percentage of CD107a− IFNγ+ PRF− TNFα+ cells was observed, while the percentage of CD107a− IFNγ− PRF− TNFα− cells tended to be lower after coculture with both DCs (Figure 5D). Therefore, in vitro coculture experiments indicated that the interaction of CD8+ T cells with TLR‐stimulated pDCs and CD141+ mDCs induces a higher and more polyfunctional SEB‐specific and HIV‐specific CD8+ T cell response.

### 2.6 PD‐1 Blockade Further Increases the CD8+ T Cell Response after TLR Stimulation in HD's Tonsils and PLWH

**FIGURE 6:**
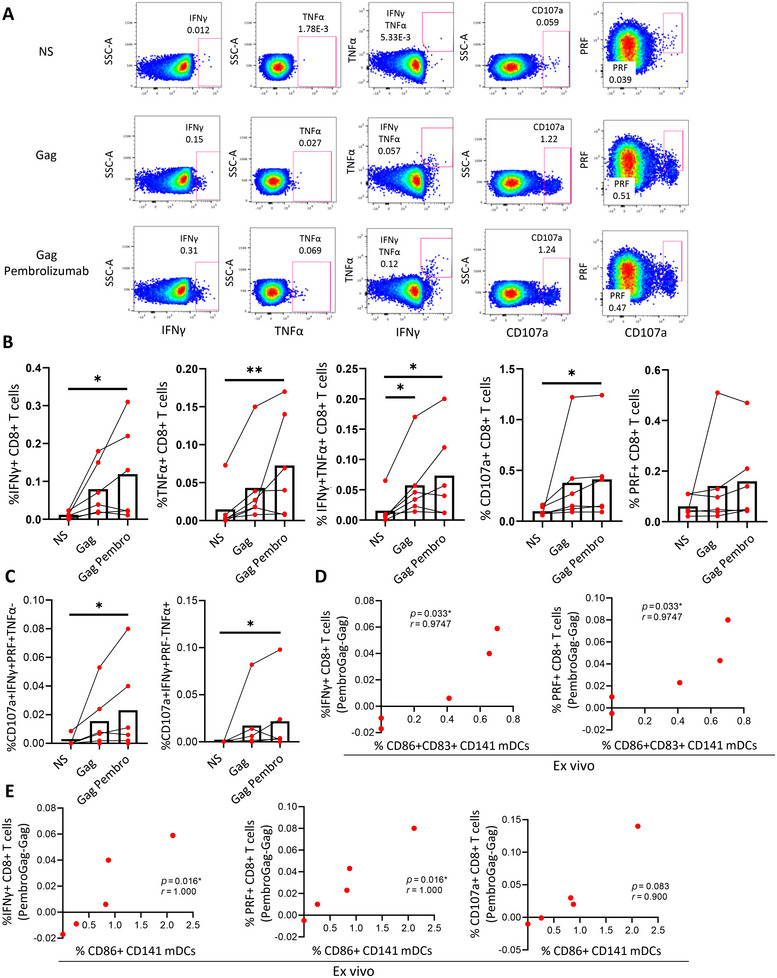
PD‐1 blockade‐mediated the increase of CD8+ T cell response after TLR stimulation in peripheral blood of PLWH. (A) Representative pseudocolor dot plots and (B) bar graphs showing the percentages of HIV Gag‐specific IFNγ+, TNFα+, IFNγ+TNFα+, CD107a+, and perforin (PRF)+ CD8+ T cells in ART naïve PLWH, after unfractionated cells stimulation with TLR agonists, in the presence and absence of pembrolizumab (Pembro). (C) Bar graphs showing the percentage of CD8+ T cells expressing different combinations of studied functions (CD107a, IFNγ, PRF, and TNFα) in ART naïve PLWH, after unfractionated cells stimulation with TLR agonists, in the presence and absence of pembrolizumab. Correlations between the percentages of HIV Gag‐specific IFNγ+, perforin (PRF)+ and CD107a+ CD8+ T cells after PD‐1 blockade in vitro, with ex vivo CD86+CD83+ (D) and CD86+ (E) CD141+ mDC percentages, in ART naïve PLWH. Each dot represents a participant. Friedman and Spearman tests were used (*n* = 7) and ROUT method was utilized to identify and discard outliers (*Q* = 0.1%). *p < 0.05, **p < 0.01, ***p < 0.001.

Ex vivo immunophenotyping of LN from PLWH showed a positive correlation between PD‐L1 expression on pDCs and PD‐1 expression on CD8+ T cells and viral load (Figure 1C,D). Moreover, PD‐1 is an immune checkpoint inhibitor, which decreases antigen‐specific CD8+ T cell response through the interaction with its ligand PD‐L1. Therefore, the last step of this study was to block the PD‐1 receptor using pembrolizumab, a PD‐1 blocking antibody that has been previously used in the clinic mainly for cancer immunotherapy (European Medicines Agency, EMA/431721/2023 and EMEA/H/C/003820), to investigate its potential along with TLR agonists as a future immunotherapeutic strategy for PLWH. Hence, unfractionated cells from blood of HD and PLWH, as well as from HD tonsils were prestimulated with TLR‐7 and TLR‐3 agonists and then cultured with the specific antigens (SEB or HIV Gag peptides) in presence or absence of pembrolizumab for PD‐1 blockade (Table S1 and Figure S5A). In this case, the experiments were carried out using unfractionated cells to simulate physiological conditions. Tonsil cells were included to determine if the same effect observed in lymphoid tissue was present in peripheral blood, given the expected higher PD‐1 expression in tissue. In fact, CD8+ T cells from blood of HD displayed a lower basal PD‐1 expression comparing with the ones from blood of PLWH or tonsils (Figure S5B). Probably for this reason, we found no difference after the PD‐1 blockade in blood from HD (Figure S5C). In contrast, when we studied PLWH, using samples from the same ART naïve participants analyzed in the ex vivo flow cytometry (*n* = 6; Table S1), we observed that PD‐1 blockade increased HIV‐specific CD8+ T cell cytokine production (IFNγ and TNFα) and degranulation (CD107a) in four out of six participants (Figure 6A,B). In addition, we observed a higher percentage of HIV‐specific CD8+ T cells producing three markers simultaneously (CD107a+ IFNγ+ PRF+ TNFα− and CD107a+ IFNγ+ PRF− TNFα+) in PLWH after PD‐1 blockade (Figure 6C). We did not observe any significant difference in the polyfunctionality when we analyzed the pie charts (Figure S5D).

To elucidate the possible factors implicated in the variability of PD‐1 blockade response among PLWH, we correlated HIV‐specific CD8+ T cell response after pembrolizumab treatment with participants’ clinical data (viral load, time since HIV diagnosis, CD4+ and CD8+ T cell counts and nadir CD4+) and all the studied DC parameters. Although some trends were found regarding CD4+ T cell counts and CCR7 and IDO expression on pDCs (data not shown), no significant associations were found, except in CD141+ mDCs. In fact, PLWH who showed increased HIV‐specific CD8+ T cell response after PD‐1 blockade, also displayed higher activated/mature CD141+ mDC percentages ex vivo. In fact, in blood of PLWH, the percentages of CD86+CD83+ CD141+ mDCs and CD86+ CD141+ mDCs ex vivo, were associated with a higher IFNγ production and PRF expression on CD8+ T cells after PD‐1 blockade in vitro (Figure 6D,E). The same tendency was observed with CD107a expression (Figure 6E). Higher CD8+ T cell IFNγ and TNFα production after pembrolizumab treatment was also positively correlated with the PD‐1 expression on fCD8 and PD‐L1, costimulatory molecules (CD83 and CD86) and homing receptors (CCR5, CCR2, CCR9) on CD141+ mDCs in LN of PLWH (Figure S5E). Regarding pDCs, increased CD8+ T cell response after PD‐1 blockade was positively correlated with CCR7 and CCR9 expression, while it was negatively correlated with IDO expression in LN of PLWH (Figure S5E).

**FIGURE 7:**
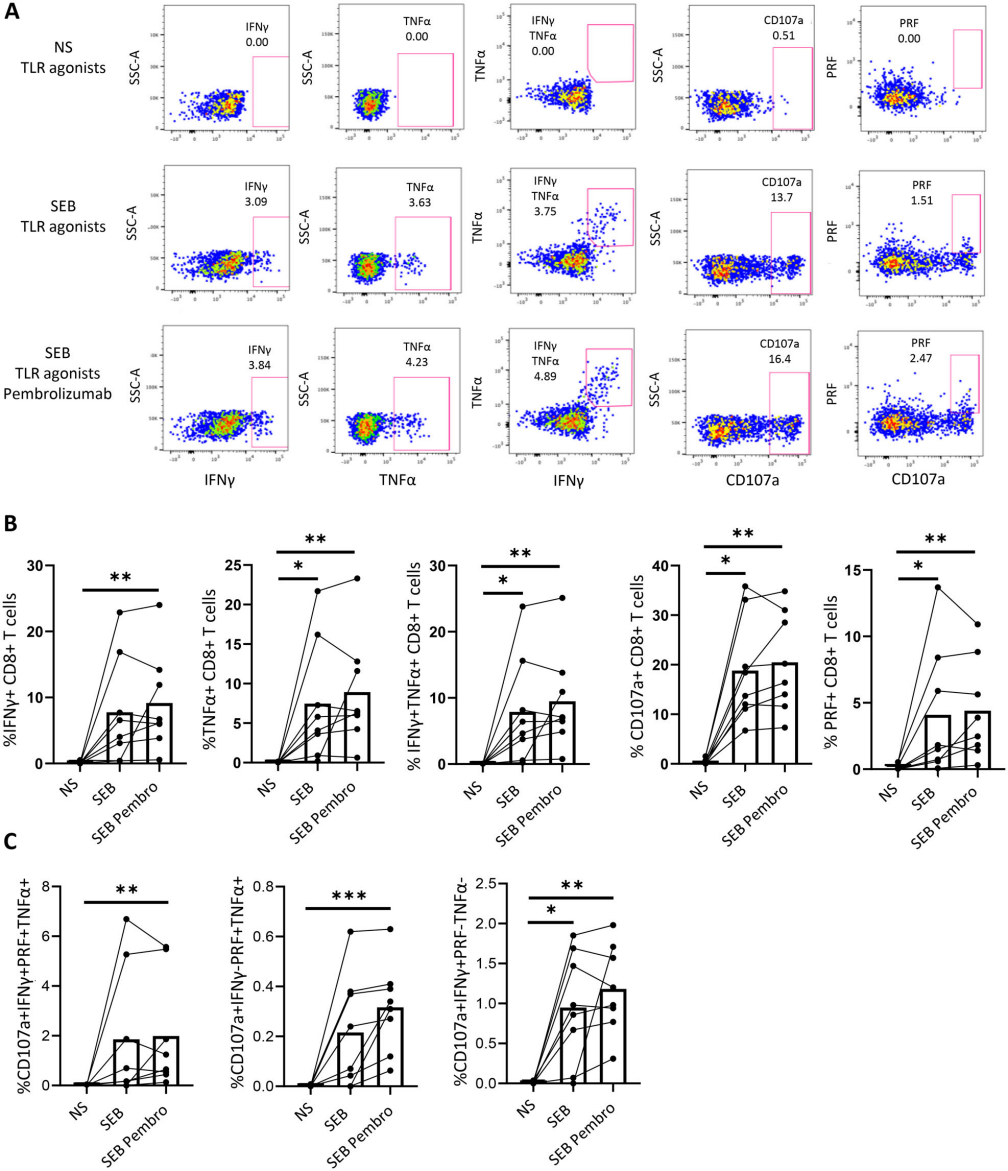
PD‐1 blockade‐mediated increase of CD8+ T cell response after TLR stimulation in HD's tonsils. (A) Representative pseudocolor dot plots and (B) bar graphs showing the percentages of SEB‐specific IFNγ+, TNFα+, IFNγ+TNFα+, CD107a+, and perforin (PRF)+ CD8+ T cells in HD tonsils, after unfractionated cells stimulation with TLR agonists, in the presence and absence of pembrolizumab (Pembro). (C) Bar graphs showing the percentage of CD8+ T cells expressing different combinations of studied functions (CD107a, IFNγ, PRF, and TNFα) in HD tonsils, after unfractionated cells stimulation with TLR agonists, in the presence and absence of pembrolizumab. Each dot represents a participant. Friedman test was used (*n* = 8) and ROUT method was utilized to identify and discard outliers (*Q* = 0.1%). *p < 0.05, **p < 0.01, ***p < 0.001.

When we studied the effect of pembrolizumab in tonsils from HD, a higher variability was observed among the subjects and lower differences were found between cells with and without PD‐1 blockade, although the tendency was the same. In fact, higher SEB‐specific CD8+ T cell response was observed in five out of eight participants after the addition of pembrolizumab (Figure 7A,B). Furthermore, we found a higher percentage of SEB‐specific CD8+ T cells in tonsils expressing four (CD107a+ IFNγ+ PRF+ TNFα+), three (CD107a+ IFNγ− PRF+ TNFα+) or two (CD107a+ IFNγ+ PRF− TNFα−) markers simultaneously (Figure 7C) after the incubation with pembrolizumab. Overall, PD‐1 blockade using pembrolizumab further increased the magnitude and polyfunctionality of the antigen‐specific CD8+ T cell response after TLR‐3 and TLR‐7 stimulation in blood of PLWH and in healthy lymphoid tissue in more than half of the participants.

## 3 Discussion

In this study, we determined the numbers, phenotype, and location of pDCs and CD141+ mDCs in LNs of PLWH. For the first time in human, we found an interaction between pDCs and CD141+ mDCs with CD8+ T cells in LNs. Additionally, the DC frequency and their interaction with CD8+ T cells were associated with participants’ viral levels in blood and tissue. The effect of this interaction in the antiviral response was demonstrated with in vitro experiments, showing a higher SEB‐ and HIV‐specific CD8+ T cell response after the coculture with TLR‐primed pDCs and CD141+ mDCs. Last, we showed increased antigen‐specific CD8+ T cell response after PD‐1 blocking using pembrolizumab along with TLR agonists.

pDCs and CD141+ mDCs are key participants in promoting an efficient antiviral T cell response by interacting with T cells to secrete cytokines and through several costimulatory molecules [8, 9]. DC–CD8+ T cell interactions occur in lymphoid organs as LNs. In a healthy human LN, pDCs are located in the T cell zone and interfollicular areas, closely associated with high endothelial venules, while CD141+ mDCs can predominantly be found within the T cell zone [27, 28]. In PLWH, pDCs are principally located in interfollicular areas [29], suggesting that pDCs tend to be closer to the follicles during HIV infection. In fact, HIV infection is known to cause follicular atrophy and the redistribution of several immune cells, including DCs [30]. Accordingly, our analysis of LNs from PLWH by confocal microscopy revealed that CD141+ mDCs were predominantly found in T cell zone, while pDCs were outside and inside the follicles. Image analysis also showed an interaction of CD8+ T cells with pDCs and CD141+ mDCs in LN of PLWH. The interaction with pDCs was closer to the center of the follicle in comparison with the interaction with CD141+ mDCs, which is in accordance with the distribution of these DC subsets across the tissue. Our results from ex vivo flow cytometry suggest that this interaction might be occurring between specific pDC and CD141+ mDC subsets. In fact, we observed an association between CD2+ CD5+ CD81+ pDCs and fCD8 in LNs from PLWH. This pDC subset has been described as a strong inducer of T cell proliferation in human blood, bone marrow, and tonsils [24]. Here, we demonstrated the presence of this specific pDC subset in LN of PLWH and its association with CD8+ T cell frequency. On the contrary, apoptotic pDCs were negatively correlated with fCD8 and CD141+ mDCs; in line with previous findings describing that pDC from PLWH with active viraemia displayed higher levels of apoptosis and T regulatory‐inducing molecules [31]. In fact, it is known that pDCs can be infected by HIV and therefore their survival and function are affected [32]. We also observed a correlation between the numbers of fCD8 with CD83+ CD141+ mDCs, being CD83 a costimulatory molecule with a key role in T cell proliferation [25]. Thus, the interaction of CD8+ T cells with these specialized DCs might be inducing CD8+ T cell function, proliferation, and/or differentiation to fCD8 in LN from PLWH.

Ex vivo flow cytometry also showed a relation between CD141+ mDCs and pDC survival, and CD141+ mDC activation and/or maturation with pDC homing to LNs; suggesting that both DC subsets might be interacting with each other. In fact, in one of the four studied LNs by confocal microscopy, we found triple interactions that included CD8+ T cells, pDCs and CD141+ mDCs. As Brewitz et al. [23] demonstrated in the murine model infected with vaccinia virus, we postulate that this interaction might be promoted by antigen‐activated CD8+ T cells, that recruit pDCs and CD141+ mDCs to the infected sites through specific chemokine secretion. This active colocalization of DCs supports their cooperation, enhancing the antigen‐specific CD8+ T cell proliferation and/or function [23]. Of note, this triple interaction was only observed in the participant with the higher viral load, and the DC–CD8+ T cell interaction occurred closer to the follicles in PLWH with higher viral loads. In this line, a previous study associated pDC redistribution and clustering with a worse HIV prognosis, specifically, with lower CD4+ T cell counts and advanced HIV‐lymphadenitis stage [29]. An explanation might be that the higher HIV load, the higher DC–CD8+ T cell interactions are required in order to control the infection. In fact, although we cannot infer causality due to the cross‐sectional study design, based on previous data in the murine model infected with vaccinia virus [23], this interaction is involved in the resolution of the acute viral infection. In the human HIV model this interaction might intend to resolve the infection and the balance finally might tip in favor of the virus. This could also be the reason why pDC–CD8+ T cell interaction occurs closer to the follicles in PLWH with high viral load, considering that HIV‐infected cells are usually concentrated in the follicles [33, 34, 35]. In contrast, we also observed by flow cytometry that pDC and CD141+ mDC frequencies in blood, as well as pDC numbers in LN, were inversely correlated with viral load. This might be indicative of the beneficial role of these DC subsets in HIV control, as we and others have previously described [5, 6, 11, 12].

The antiviral effect of pDC–CD141+ mDC–CD8+ T cell interaction was demonstrated by in vitro coculture experiments. The cooperation of both primed pDCs and CD141+ mDCs enhanced SEB‐specific and HIV‐specific CD8+ T cell response in HD and PLWH, respectively. This fact indicates that the interaction of pDC and CD141+ mDCs with CD8+ T cells might occur in response to different antigens/pathogens, not only in the context of HIV infection. The interaction with both DC subsets specially affected IFNγ and TNFα cytokine production. In terms of cytotoxic capacity (degranulation and perforin) or polyfunctionality, no significant differences were found between the interaction with only pDCs or with both pDCs and CD141+ mDCs, suggesting a more relevant contribution of pDCs in the T cell priming in this context. Moreover, TLR prestimulation of DCs did not affect the CD8+ T cell cytotoxic capacity, but clearly had an effect in cytokine production. It might be hypothesized that the presence of other immune cells might be required to observe an effect in the CD8+ T cell cytotoxic capacity after TLR‐mediated DC stimulation. Regarding the interaction with only CD141+ mDCs, a marginal no significant increase of SEB‐specific CD8+ T cell response was found. An explanation may be that low CD141+ mDC numbers were added to the coculture. These cell numbers were chosen based on the proportion of this cell type in physiological conditions. However, it could be hypothesized that increasing the number of CD141+ mDC or expanding them might lead to a more effective antigen‐specific CD8+ T cell priming. In fact, Domenjo‐Vila et al. [13] demonstrated that in a LCMV mouse model, the expansion of XCR1+ mDCs improved viral control. Of note, in spite of the lack of effectiveness for CD8+ T cell priming by themselves, CD141+ mDCs had a synergic effect along with pDCs, even at low numbers, supporting the fact that there is an interaction between the three cell types.

In our experimental design, TLR‐7 and TLR‐3 prestimulation by GS‐9620 and Poly I:C respectively, simulate pDC and CD141+ mDC priming by pathogen antigens, but additionally, highlights the potential of TLR agonists as immunomodulatory agents. Our results showed that DC prestimulation by these TLR agonists was important for an efficient pDC‐ and CD141 mDC‐dependent induction of antigen‐specific CD8+ T cell response. In fact, our group previously described the capacity of different TLR agonists to induce pDC‐mediated increase of HIV‐specific CD8+ and CD4+ T cell responses [6]. In addition, thanks to their capacity to induce both innate and adaptive immune activation, TLR agonists have been used in several clinical trials as immunotherapy to improve antiviral response and as latency reversal agents in the context of HIV/SIV infection, including the TLR‐7 agonist GS‐9620, also known as vesatolimod [3, 4, 14, 15, 36, 37]. Similarly, TLR‐3 agonists have shown promising results both in vitro and in vivo as potential vaccine adjuvants, capable of potentiating DC abilities to induce HIV‐1‐specific CD8+ T cell responses [12, 38]. TLR agonists have also been utilized in combination with other compounds, including broadly neutralizing antibodies or different vaccines [7, 36, 37, 39]. Our results reveal the potential of TLR agonists along with PD‐1 blocking strategies for the induction of antiviral response. Despite the variability, more than 50% of the participants showed a higher HIV‐specific CD8+ T cell response when PD‐1 was blocked by pembrolizumab after TLR‐3 and TLR‐7 prestimulation. In this line, a recent work reported that mRNA vaccine prototypes TMEP‐B and TMEP‐Bmod, when combined with the TLR‐7 agonist vesatolimod and/or PD‐1 blocking antibody nivolumab, enhanced in vitro HIV‐specific T cell responses [7]. Unlike the DC–CD8+ coculture experiment in PLWH, PD‐1 blocking induced both cytokine production and degranulation capacity of CD8+ T cells; an explanation might be that as this experiment was carried out with unfractionated cells, other immune cell types might have participated in this process. The enhanced CD8+ T cell antiviral response induced by this combined therapy probably would promote the elimination of latently infected cells, decreasing HIV reservoir. Although the total elimination of HIV reservoirs, achieving a sterilizing cure, is very challenging, an improvement in anti‐HIV immunity by this type of immunotherapies might reach long‐term virological remission or functional cure in PLWH. However, although pembrolizumab along with TLR agonists previously showed a manageable safety profile in cancer [40], further studies would be required to determine not only the effectiveness but also the possible side effects of this therapy in PLWH.

As explained above, variability in PD‐1 blockade response was observed among PLWH. This variability might be associated with specific clinical and/or immunological characteristics of each participant. When we correlated CD8+ T cell response to pembrolizumab with clinical data and studied pDC parameters in our cohort of PLWH, we did not find any significant association. However, we observed that this variability in PD‐1 blockade observed in PLWH was associated with a higher frequency of mature/activated CD141+ mDCs ex vivo, underlining the potential relevance of this DC subset in this scenario. In fact, Domenjo‐Vila et al. [13] demonstrated in a LCMV murine model that XCR1+ DCs are crucial for the success of checkpoint inhibitor‐based therapies by activating different exhausted CD8+ T cell subsets. In addition to TLR agonists, pembrolizumab has also been proposed as a latency reversal agent. In fact, PD‐1/PD‐L1 axis had a relevant role in the restriction of HIV transcription and PD‐1 blocking using pembrolizumab reversed HIV latency in vivo [41, 42]. The study's variable response to PD‐1 blockade raises the possibility of using personalized medicine techniques. Given that individuals with greater percentages of activated/mature CD141+ mDCs responded better to pembrolizumab, this could be used as a biomarker to determine which patients are most likely to benefit from this treatment. Moreover, activation or expansion of CD141+ mDCs using TLR agonists or similar compounds prior immune check‐point blockade might improve the effectiveness of this type of immunotherapies, which might be relevant, not only as a potential antiviral strategy, but also as cancer immunotherapy, including adoptive transfer strategies using this cell type.

During the study, we dealt with some challenges, being the most limiting one the sample availability. Hence, a priority order was established for the development of the experiments and flow cytometry panels, that is why the sample size was not the same among the different assays and panels. This fact made unfeasible to carry out the in vitro experiments with cells from LN. However, we performed some of the in vitro experiments with cells from another lymphoid tissue, specifically tonsillar cells. In addition, the coculture experiments required a very high number of freshly isolated cells to obtain CD141+ mDCs, which is a very rare subset. This also limited our sample size, mainly in experiments with PLWH; nonetheless, significant differences were found. For this reason, due to fresh sample management, participants from a single hospital were included, which may limit applicability to larger and more diverse populations and we could not make adequate comparisons between PLWH on ART and ART naïve people. Thus, further studies with a larger cohort are required for a more in‐depth examination of how ART might affect these DC–CD8+ T cell interactions. In addition, the cross‐sectional design of the present study, limits the ability to infer causality or dynamic changes over time in DC and T cell interactions; nevertheless, the wide distribution of viral load of the participants may recapitulate different stages of HIV‐disease progression in a cross‐sectional design. In this line, further studies might be required to investigate these cell interactions and the effect of the proposed therapy with a PD‐1 blocking agent and TLR agonists in particular groups of PLWH with different comorbidities and clinical characteristics as immunological nonresponders vs responders, or HIV elite controllers vs ART‐controllers. Last, another limitation was the lack of an isotype control antibody for PD‐1 blockade assay; however, we do not expect high levels of unspecific Fc‐Receptor‐mediated stimulation.

In summary, we demonstrated an interaction between pDCs, CD141+ mDCs and CD8+ T cells in blood and LN from PLWH, which improves HIV‐specific CD8+ T cell response and is associated with viral levels in blood and tissue. These results show the importance of the cooperation of different DC subsets to achieve a more potent anti‐HIV adaptive response and support the use of DC‐based immunotherapies for HIV control. This study also reveals the potential use of TLR agonists alone or in combination with PD‐1 blockade as an immunotherapeutic tool for enhancing the antiviral response in HIV cure strategies.

## 4 Material and Methods

### 4.1 Participants and Samples

PLWH (*n* = 14) were recruited consecutively at the Clinic Unit of Infectious Diseases, Microbiology and Parasitology at Virgen del Rocío University Hospital (HUVR, Seville, Spain). Twelve were naïve for ART with detectable viremia in plasma (>20 HIV‐RNA copies/mL). Two PLWH on ART with undetectable viral load (<20 HIV‐RNA copies/mL) were also included exclusively for DC–CD8+ T cell coculture experiments (Table S1). Participants were negative for hepatitis C and B virus exposure, were not under any immunosuppressive or immunomodulatory treatment and did not have any other clinically active infection. Peripheral blood (*n* = 14) and inguinal LNs (*n* = 8) were obtained from PLWH. Whole LNs were extracted after minor surgery and previous echography to check LN location. Due to the small size of the LNs, priority was given to ex vivo flow cytometry (*n* = 8), and confocal microscopy was performed with remained material (*n* = 4) (Table S1). Clinical data of PLWH are described in Table S1. Anonymized blood samples of HD were provided by the Regional Center for Blood Transfusion and Tissue Bank Sevilla‐Huelva (Seville, Spain). Tonsils were obtained from anonymized discarded pathological specimens from otorhinolaryngology department at HUVR. The study was approved by the Ethics Committee of the HUVR (Study Codes: 1594‐N‐17, 2013PI/115). All subjects provided written and signed informed consent in accordance with the Declaration of Helsinki.

### 4.2 Ex Vivo Flow Cytometry

First, DCs and T cells from peripheral blood and LNs of PLWH were analyzed by ex vivo multiparametric flow cytometry. Peripheral blood mononuclear cells (PBMCs) were obtained by density gradient (CPT tubes; BD Biosciences) and LN cells by mechanic disruption and filtration in cold RPMI medium. For DC and T cell phenotyping, cells were stained with a viability marker (Zombie Aqua Fixable Viability Kit from BioLegend for DC panels; LIVE/Dead Fixable Aqua from Invitrogen for T cell panel) and an apoptosis marker (BUV395 Annexin V from BD Biosciences for pDC panel) following manufacturer's instructions. Then, cells were stained with fluorochrome‐conjugated antibodies for 30 min at 4°C and fixed and permeabilized with Fixation/Permeabilization Buffer Set (eBioscience) following manufacturer's protocol to detect intracellular markers (TLR‐9 and IDO). Samples were acquired with a FACSymphony A5 Flow Cytometer using FACSDiva software (BD Biosciences). Data were analyzed utilizing FlowJo v9.2 software. Fluorochrome‐conjugated markers are indicated in Table S2 and the gating strategy in Figure S1A,B. CD4+ and CD8+ T cell subsets were analyzed as: naïve (CD45RO− CD27+), central memory (CM; CD45RO+ CD27+), effector memory (EM; CD45RO+ CD27−), and terminal differentiated effector memory (TEMRA; CD45RO− CD27−) (Figure S1A). TFH (CXCR5+ PD1+) and fCD8 (CXCR5+ CCR7−) were identified as previously described [19, 43] (Figure S1A). pDCs were identified as Lin2− CD123+ BDCA2+ and mDCs as Lin2− HLA‐DR+ CD11c+; within mDCs, CD1c+, CD141+, and CD16+ subsets were selected (Figure S1B). All gates were stablished based on negative cell subsets for the marker of interest. For these experiments, a priority order was established depending on sample availability: (1) pDC panel, (2) DC panel and (3) T cell panel. However, as lower cell numbers were required for the T cell panel (at least 2 × 10^5^ cells) than the DC panels (at least 1 × 10^6^ cells), only T cells were analyzed in some LN samples in which the sample was insufficient for pDC and DC panels (LN: pDC panel *n* = 6, DC panel *n* = 5, T cell panel *n* = 7; blood: pDC panel *n* = 7, DC panel *n* = 6, T cell panel *n* = 7).

### 4.3 LN Immunofluorescence

LNs were harvested and fixed with PLP buffer containing 0.2 M l‐lysine (pH 7.4), 2 mg/mL NaIO_4_, and 4% of paraformaldehyde (PFA) in 0.1 M phosphate buffer (PBS) overnight at 4°C. Then, LNs were immersed in 30% sucrose solution for another 24 h and were frozen in optimal cutting temperature compound and stored at −80°C. 10 µm sections were then sliced with a cryostat (Leica CM1950). Immunofluorescence analyses were carried out as previously described [22, 26]. Briefly, LN sections from PLWH were incubated in blocking solution (1% bovine serum albumin [Invitrogen], 0.2% v/v Triton X‐100 [Sigma–Aldrich, Merck] in PBS) for 1 h at room temperature (RT) and then, stained with the following primary antibodies overnight at 4°C: rabbit anti‐CLEC9a (1:10; ThermoFisher), mouse IgG2a anti‐CD11c (1:10, clone 2F1C10; Proteintech), mouse IgG1 anti‐CD123 (1:5, clone BSB.59; Bio SB) and mouse IgG2b anti‐CD8 (1:150, clone 4B11; Invitrogen). LN sections were then incubated with secondary antibodies for 2 h at RT: donkey anti‐rabbit IgG BV421 (1:250, clone Poly4064; BioLegend), goat anti‐mouse IgG2a Alexa Fluor 488 (1:250, polyclonal; Thermo Scientific), goat anti‐mouse IgG1 Alexa Fluor 546 (1:250, polyclonal; Thermo Scientific), and goat anti‐mouse IgG2b Alexa Fluor 647 (1:250, polyclonal; Thermo Scientific). Last, they were incubated with fluorochrome‐conjugated antibodies for 2 h at RT: anti‐CD20 eFluor 615 (1:20, clone L26; Invitrogen) and anti‐CD4 Alexa Fluor 700 (1:5, polyclonal; R&D Systems). On the other hand, p24 staining was performed in three LN samples. After 1 h of blocking, samples were incubated with anti‐CD20 eFluor 615 (1:20, clone L26; Invitrogen), anti‐CD4 Alexa Fluor 700 (1:5, polyclonal; R&D Systems), and anti‐p24 FITC (1:100, clone KC57; Beckman Coulter) for 2 h at RT. In both cases, samples were incubated with the nuclear marker JOPRO‐1 (1:15,000; Invitrogen) for 25 min at RT and then mounted with Fluoromount‐G (Invitrogen) for visualization.

### 4.4 Image Acquisition, Analysis, and Visualization

Images were taken using a Leica Stellaris 8 laser‐scanning confocal microscope, HC PL FLUOTAR 20x/0.75 W.D. 0.67 nm and/or HCX PL APO 40x/0.95 W.D. 0.17 nm objectives. All acquisition parameters were kept constant and taken by the same researcher, blinded to the viral load. The fluorescently labeled structures were analyzed using Fiji Image J Software (W. Rasband; NIH) and Imaris software (v.9.6; Oxford Instruments) and represented as maximum intensity projections and 3D rendering images, respectively. Histocytometry analyses were carried out as previously described [18, 22]. pDCs were identified as CD11c− CD123+ and CD141+ mDCs as CD11c+ CLEC9a+. For CD8+ T cell identification, cells with high expression of CD8 were selected. For DC panel, we first set up the follicle based on the CD20 expression and then, the distance between the center of the follicle and each DC–CD8+ T cell interaction was analyzed using the straight tool (Fiji ImageJ software) (Figure 2D). To analyze the colocalization of CLEC9a–CD8 and CD123–CD8, “surface” and “coloc” plug‐ins of Imaris software were used. Data were represented as Manders coefficients [44]. For p24 staining, image background was subtracted based on the expression of this marker in non‐HIV tissue. Using the Imaris software, images were automatically reconstructed into a 3D model and the percentage of p24+ cells was calculated.

### 4.5 Cell Isolation and Sorting

PBMCs from HD and PLWH were isolated from blood by density gradient, using Ficoll‐Paque (Lymphoprep; StemCell Technologies) or CPT tubes (BD Biosciences), respectively. For the isolation of each subpopulation of interest, autologous CD8+ T cells and total DCs were separated by negative selection using commercial kits (EasySep Human Pan‐DC Pre‐Enrichment Kit and EasySep Human CD8+ T cell Enrichment Kit, from StemCell). Around 25 × 10^6^ and 200 × 10^6^ of PBMCs were used for CD8+ T and total DC isolation, respectively. For pDC and CD141+ mDC sorting, total DCs were stained for 5 min at RT with a viability marker (LIVE/DEAD Fixable Aqua Dead Cell Stain, Life Technologies) and 10 min at RT with the following fluorochrome‐conjugated antibodies: Lin‐2 FITC (CD3, CD14, CD19, and CD56), anti‐HLA‐DR BV711 (clone G46‐6) and anti‐CD11c BV650 (clone B‐ly6) from BD Biosciences; anti‐CD123 Alexa Fluor 700 (clone 32703) from R&D Systems; and anti‐CD141 PE‐Cy7 from BioLegend. Stained DCs were washed with PBS (5 min, 1800 rpm, RT) and resuspended in 1 mL of 2% fetal bovine serum (FBS) in PBS. pDCs (Lin‐2− HLA‐DR+ CD11c− CD123+) and CD141+ mDCs (Lin‐2− HLA‐DR+ CD11c+ CD141+) were sorted using the Cell Separator FACS Aria Fusion (Becton Dickinson) and collected in RPMI with 20% of FBS. In all cases, the purity was higher than 90% (Figure S6A).

### 4.6 DC–CD8+ T Cell coculture

Coculture experiments with autologous cells were carried out using a ratio 1 (CD141+ mDCs):10 (pDCs):100 (CD8+ T cells) in 96 well‐plates in R10 medium (RPMI 1640 supplemented with 10% FBS and 1% penicillin‐streptomycin and L‐glutamine). First, CD141+ mDCs and pDCs were prestimulated for 18 h at 37°C with 2 µg/mL of TLR‐3 agonist Poly I:C (InvivoGen) and 10 ng/mL of TLR‐7 agonist GS‐9620 (Cayman Chemical), respectively. CD8+ T cells were maintained at 4°C during DC prestimulation. Then, after a resting of 30 min at 37°C, CD8+ T cells were added to the cell culture without washing, and incubated along with DCs for 6 h at 37°C. The following experimental conditions were included: CD8+ T cells alone, CD8+ T cells with only pDCs or CD141+ mDCs, and CD8+ T cells with both pDCs and CD141+ mDCs. This coculture was done in the absence or presence of 1 µg/mL of SEB (Sigma–Aldrich, Merck) for HD, and 2 µg/peptide/mL of HIV Gag peptides pool (HIV Reagent Program, ARP‐12425) for PLWH. All cells were incubated with anti‐CD107a BV605 (clone H4A3; BioLegend), monensin (Golgi Stop; BD Biosciences), and brefeldin A (Golgi Plug; BioLegend) following manufacturer's instructions. A schematic representation of the followed protocol is indicated in Figure S6B.

### 4.7 PD‐1 Blockade Assay

Frozen PBMCs from HD and PLWH, and frozen cells from HD tonsils were in vitro stimulated. PBMCs were isolated as described above and cells from tonsils were obtained by mechanic disruption and filtration in cold RPMI medium. Cells were cryopreserved in freezing medium (90% of FBS + 10% dimethyl sulfoxide [PanReac AppliChem]) in liquid nitrogen until further use. Then, cells were thawed and 1.5 × 10^6^ cells resuspended in 1 mL of R10 medium. PBMCs were prestimulated with 2 µg/mL of Poly I:C and 10 ng/mL of GS‐9620 for 18 h at 37°C. Then, cells were incubated with 1 µg/mL of SEB (HD) or 2 µg/peptide/mL of HIV Gag peptides (PLWH) for 6 h at 37°C, in the presence or absence of 5 µg/mL of pembrolizumab (anti‐human PD‐1 monoclonal antibody (MedChemExpress)) for PD‐1 blockade. A condition of nonstimulated cells was included as a negative control.

### 4.8 Intracellular Cytokine Staining

After in vitro stimulation (DC–CD8+ coculture and PD‐1 blockade assays), cells were collected and washed with PBS, as described above. To determine antigen‐specific CD8+ T cell response, cells were stained for 30 min at 4°C with the same viability marker (LIVE/DEAD Fixable Aqua Dead Cell Stain; Life Technologies) and with the following fluorochrome‐conjugated antibodies for extracellular markers: anti‐CD45RA PE‐Cy7 (clone L48), anti‐CD27 BV605 (clone L126), anti‐CD8 PerCP‐Cy5.5 (clone SK1), and anti‐CD3 V450 (clone SP34‐2) from BD Biosciences. In PD‐1 blockade assay, the anti‐PD‐1 BV786 (clone EH12‐1; BD Biosciences) was also included to determine the level of PD‐1 blocking, as this antibody shares the epitope with pembrolizumab. Cells were fixed and permeabilized using Cytofix/Cytoperm Kit (BD Biosciences) following manufacturer's protocol. Last, the staining for intracellular markers was performed for 30 min at 4°C adding the following antibodies: anti‐IFNγ APC (clone B27) and anti‐TNF‐α Alexa Fluor 700 (clone MAb11) from BD Biosciences; and anti‐Perforin PE (clone B‐D48) from BioLegend. Cells were resuspended in 4% of PFA in PBS and samples were acquired with LRS II Fortessa Flow Cytometer using FACSDiva software (BD Biosciences). Data were analyzed utilizing FlowJo v10.4.2 software (BD Life Sciences).

### 4.9 Statistical Analysis

Statistical Package for the Social Sciences software (SPSS 25.0; SPSS, Inc.) and Graph Pad Prism v8.0 (GraphPad Software, San Diego, CA, USA) were used for statistical analysis. Correlations between variables were analyzed using the Spearman test. The Wilcoxon nonparametric paired test was used to analyze two groups, while Friedman and Kruskal–Wallis tests, including Dunn's multiple comparisons test correction, were applied to compare more than two groups. ROUT method was utilized to identify and discard outliers (*Q* = 0.1%). For polyfunctionality analysis, pie chart graphs and the Permutation test to assess differences between pie charts were done using Pestle v1.6.2 and Spice v6.0. All differences with a *p* value <0.05 were considered statistically significant (*p < 0.05, **p < 0.01, ***p < 0.001, ****p < 0.0001).

## Author Contributions

L.L.C. and A.M. recruited the participants, and S.F., L.E.V., and I.G. participated in blood and LN processing. E.R.M. and J.V. designed the experiments. J.V., S.B., B.D.M., E.M., M.I.C.S., A.P.G., M.R.J.L., and C.G.C. performed the experiments. J.V., S.B., and E.R.M. analyzed and interpreted the data and wrote the paper. M.R.E.I. and F.J.O. reviewed and contributed to paper discussion. R.K. and C.P. collaborated in project coordination at Vaccine Research Center (US) and paper discussion. E.R.M. conceived the idea, coordinated the project and acquired funding for the study. All authors reviewed and approved the submitted version of the manuscript.

## Ethics Statement

The study was approved by the Ethics Committee of the HUVR (Study Codes: 1594‐N‐17, 2013PI/115). All subjects provided written and signed informed consent in accordance with the Declaration of Helsinki.

## Conflicts of Interest

Authors declare that they have no conflicts of interest. Data generated by this study are available upon request to the corresponding author.

## Supporting information




**Supplementary File 1**: mco270354‐sup‐0001‐SuppMat.pdf.

## Data Availability

Due to the sensitivity of the data, individual participant data will not be made available. Data generated by this study are available upon request to the corresponding author.
