# Supplementary material for: Plasmacytoid and CD141+ Myeloid Dendritic Cells Cooperation with CD8+ T Cells in Lymph Nodes is Associated with HIV Control
Source: MedComm (2020). 2025 Sep 12;6(9):e70354. doi: 10.1002/mco2.70354 (PMC12426486; doi:10.1002/mco2.70354)
Supplement: Supplementary file 1 — mco270354‐sup‐0001‐SuppMat.pdf. [file MCO2-6-e70354-s001.pdf]

Table S1. Study participants

PLWH											
Code	Sample type	Sex	Age (years)	ART	VL	Time since HIV diagnosis (months)	Nadir CD4	CD4	CD8	CD4/ CD8 ratio	Experiments
LN05	LN Blood	male	26	No	3.06	3.6	392	392	723	0.54	ExFC, CM, InPembro
LN08	LN Blood	male	23	No	4.68	1.56	415	428	591	0.72	ExFC, InPembro
LN10	LN Blood	male	34	No	3.83	19.2	323	652	737	0.88	ExFC
LN12	LN Blood	male	36	No	5.61	6.36	333	487	1061	0.46	ExFC, CM, InPembro
LN13	LN Blood	male	31	No	4.97	23.4	312	378	918	0.41	ExFC, CM
LN14	LN Blood	male	27	No	4.65	2.16	733	752	945	0.80	ExFC, InPembro
LN15	LN Blood	male	20	No	4.02	7.13	330	574	854	0.67	ExFC, InPembro
LN16	LN Blood	male	29	No	4.73	0.96	356	376	544	0.69	ExFC, CM, InPembro
HIV2	Blood	male	36	Yes	<20	204	673	714	754	0.95	InDC-CD8
HIV7	Blood	male	47	No	5.18	0.0	414	414	512	0.80	InDC-CD8
HIV10	Blood	male	22	No	5.08	0.0	322	407	880	0.46	InDC-CD8
HIV11	Blood	male	37	No	3.95	0.0	611	611	1494	0.41	InDC-CD8
HIV13	Blood	male	29	No	3.27	3.0	562	562	894	0.63	InDC-CD8
HIV203	Blood	male	49	Yes	<20	72.5	468	998	1505	0.66	InDC-CD8

HIV seronegative		
Sample type	Sample size (n)	Experiments
HD Peripheral Blood	13	InDC-CD8 (n=13), InPembro (n=7)
HD Tonsils	8	InPembro

Abbreviations: Nadir CD4 (Nadir CD4+ T cell count (cells/mm^3)); CD4 (CD4+ T-cell count (cells/mm^3)); CD8 (CD8+ T-cell count (cells/mm^3)); VL (Viral load (log HIV-1 RNA copies/mL)), ExFC (Ex vivo Flow Cytometry), CM (Confocal Microscopy), InPembro (In vitro Pembrolizumab assay) and InDC-CD8 (In vitro DC-CD8+ co-culture assay).

Figure S1

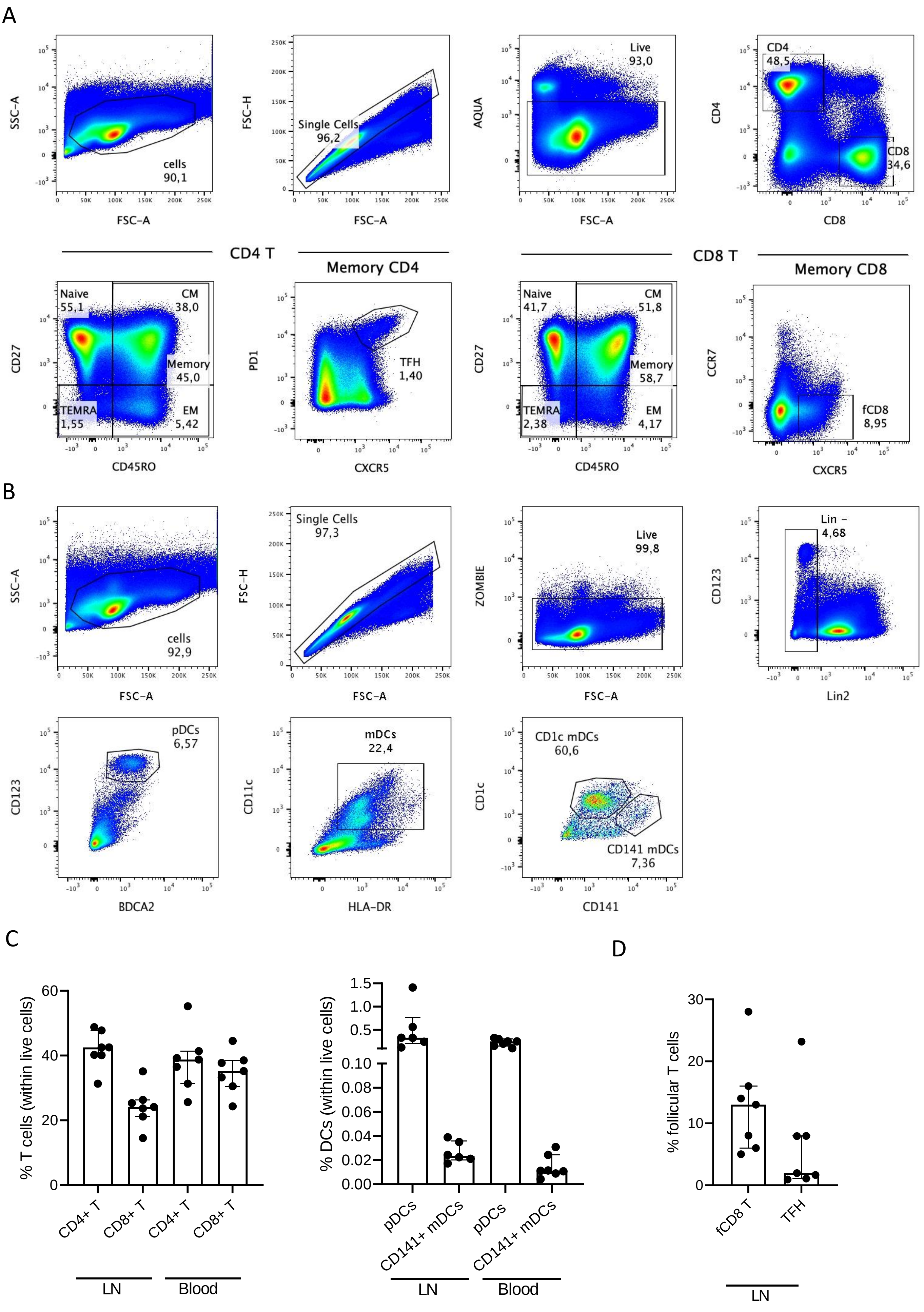

A
SSC-A
FSC-A
cells
90,1
FSC-H
Single Cells
96,2
AQUA
Live
93,0
CD4
CD8
48,5
34,6
CD4 T
Memory CD4
CD27
CD45RO
Naive
55,1
CM
38,0
Memory
45,0
TEMRA
1,55
EM
5,42
PD1
CXCR5
TFH
1,40
CD8 T
Memory CD8
Naive
41,7
CM
51,8
Memory
58,7
TEMRA
2,38
EM
4,17
CCR7
fCD8
8,95
B
cells
92,9
Single Cells
97,3
ZOMBIE
Live
99,8
CD123
Lin2
Lin –
4,68
BDCA2
pDCs
6,57
CD11c
HLA-DR
mDCs
22,4
CD1c
CD141
CD1c mDCs
60,6
CD141 mDCs
7,36
C
% T cells (within live cells)
CD4+ T
CD8+ T
CD4+ T
CD8+ T
LN
Blood
% DCs (within live cells)
pDCs
CD141+ mDCs
pDCs
CD141+ mDCs
LN
Blood
D
% follicular T cells
fCD8 T
TFH
LN

Figure S1. pDC, CD141+ mDC and T cell percentages in LN and blood. Dot plots showing the gating strategy for **(A)** follicular CD4+ and CD8+ T cells (TFH and fCD8, respectively) and **(B)** plasmacytoid (pDC) and CD141+ myeloid (mDCs) dendritic cells identification. Cells from a representative LN is shown. Bar graphs showing **(C, left)** total CD4+ and CD8+ T cell percentages within live cells, **(C, right)** pDC and CD141+ mDC percentages within live cells and **(D)** fCD8 and TFH, in LN from PLWH. Each dot represents a participant (n = 8).

Figure S2

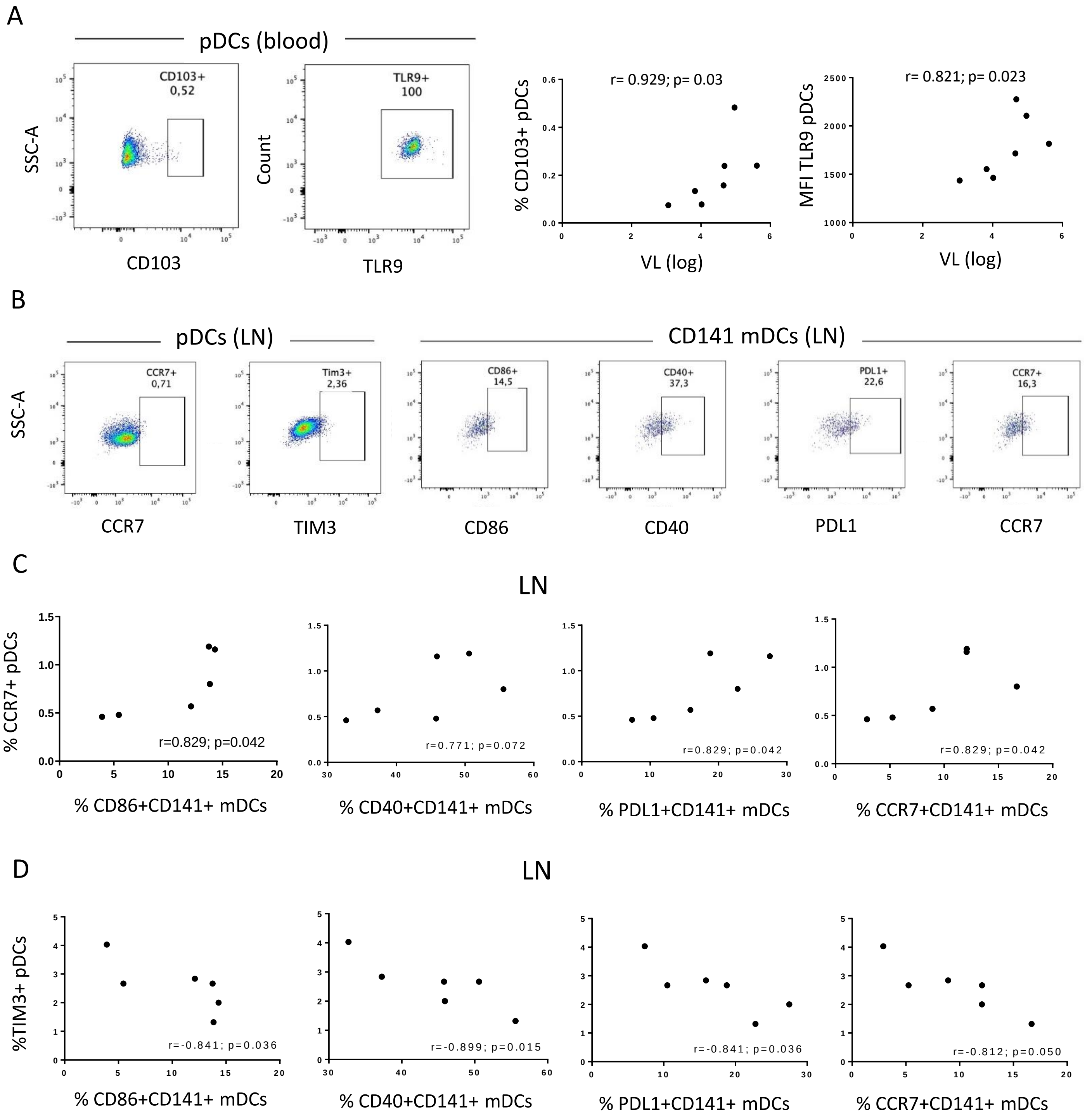
A
pDCs (blood)
CD103+
0,52
SSC-A
CD103
TLR9+
100
Count
TLR9
r= 0.929; p= 0.03
% CD103+ pDCs
VL (log)
r= 0.821; p= 0.023
MFI TLR9 pDCs
VL (log)
B
pDCs (LN)
CD141 mDCs (LN)
SSC-A
CCR7+
0,71
CCR7
Tim3+
2,36
TIM3
CD86+
14,5
CD86
CD40+
37,3
CD40
PDL1+
22,6
PDL1
CCR7+
16,3
CCR7
C
LN
% CCR7+ pDCs
r=0.829; p=0.042
% CD86+CD141+ mDCs
r=0.771; p=0.072
% CD40+CD141+ mDCs
r=0.829; p=0.042
% PDL1+CD141+ mDCs
r=0.829; p=0.042
% CCR7+CD141+ mDCs
D
LN
%TIM3+ pDCs
r=-0.841; p=0.036
% CD86+CD141+ mDCs
r=-0.899; p=0.015
% CD40+CD141+ mDCs
r=-0.841; p=0.036
% PDL1+CD141+ mDCs
r=-0.812; p=0.050
% CCR7+CD141+ mDCs

Figure S2. Correlation between pDC and CD141+ mDCs expressing activation/homing markers. (A) Gating and correlations of the percentage of pDCs expressing CD103 and TLR9 in peripheral blood with viral load (VL) in PLWH. Gating **(B)** and correlations of **(C)** the percentage of pDCs expressing CCR7 and **(D)** TIM-3 with the percentages of CD141+ mDCs positive for activation and homing markers (CD86, CD40, PD-L1 and CCR7) in LN from PLWH. Each dot represents a participant. Spearman test was used (n = 6).

Figure S3

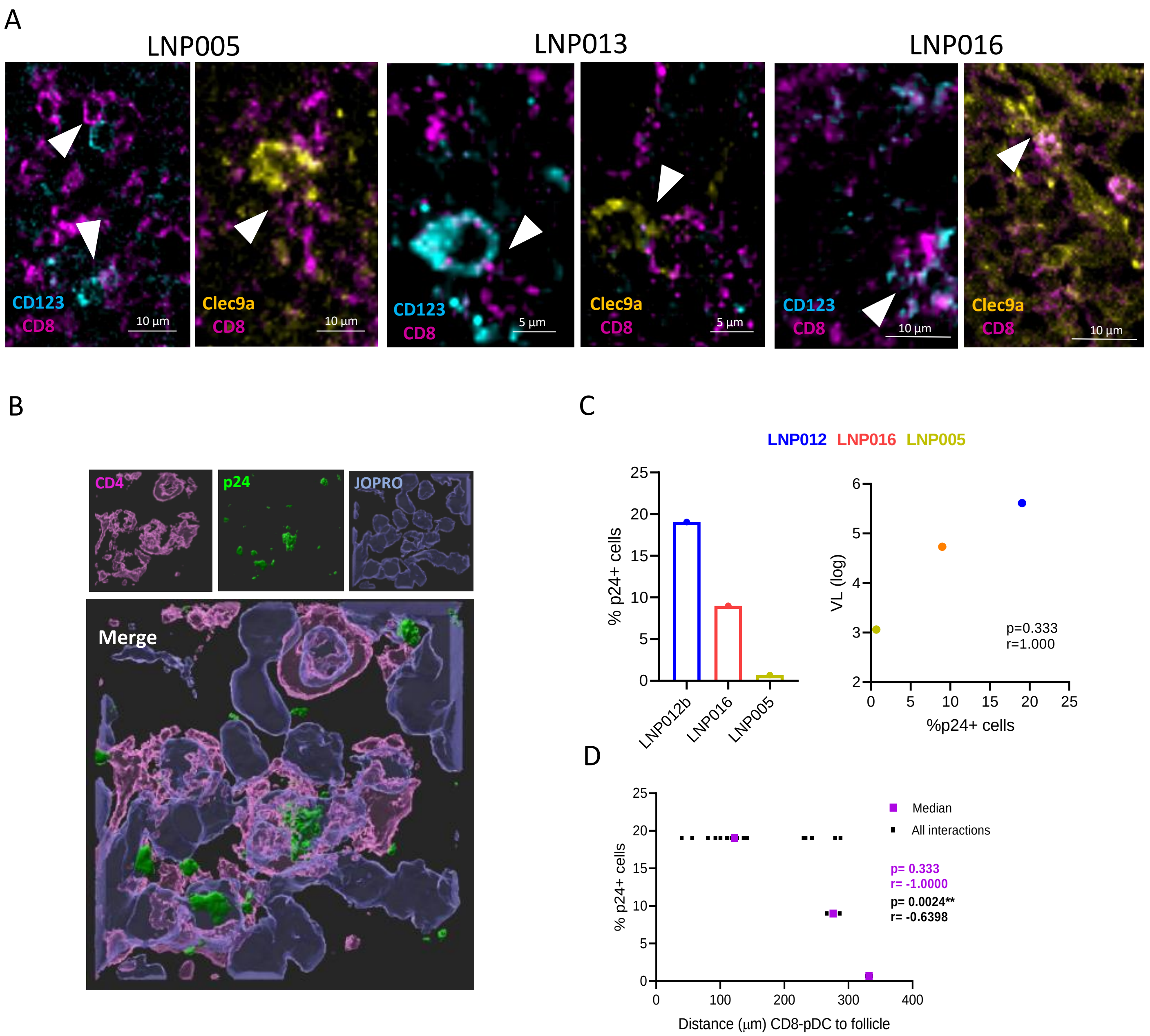
A
LNP005
LNP013
LNP016
CD123
CD8
Clec9a
10 µm
5 µm
B
CD4
p24
JOPRO
Merge
C
LNP012 LNP016 LNP005
% p24+ cells
LNP012b
LNP016
LNP005
VL (log)
p=0.333
r=1.000
%p24+ cells
D
Median
All interactions
p= 0.333
r= -1.0000
p= 0.0024**
r= -0.6398
% p24+ cells
Distance (µm) CD8-pDC to follicle

Figure S3. pDC, CD141+ mDC and CD8+ T cell interactions and HIV infection in LN. (A) Representative confocal microscopy images showing CD8+ T cell (magenta) – pDC (cyan) contact and CD8+ T cell (magenta) – CD141 mDC (yellow) contact in all the LNs from PLWH. **(B)** Representative microscopy image showing p24 staining; CD4+ T cells (magenta), p24 (green) and nucleus (JOPRO, blue). **(C)** Bar graphs showing the percentage of p24+ cells in LN of PLWH (left) and the correlation of this percentage with VL (right). **(D)** Correlation of the distance of pDC – CD8+ T cell interaction to the follicle with the percentage of p24+ cells; all interactions are showed in black and the median of the interactions in each participant are represented in purple. PLWH are represented with different colors. Spearman test was used. **P < 0.01.

Figure S4

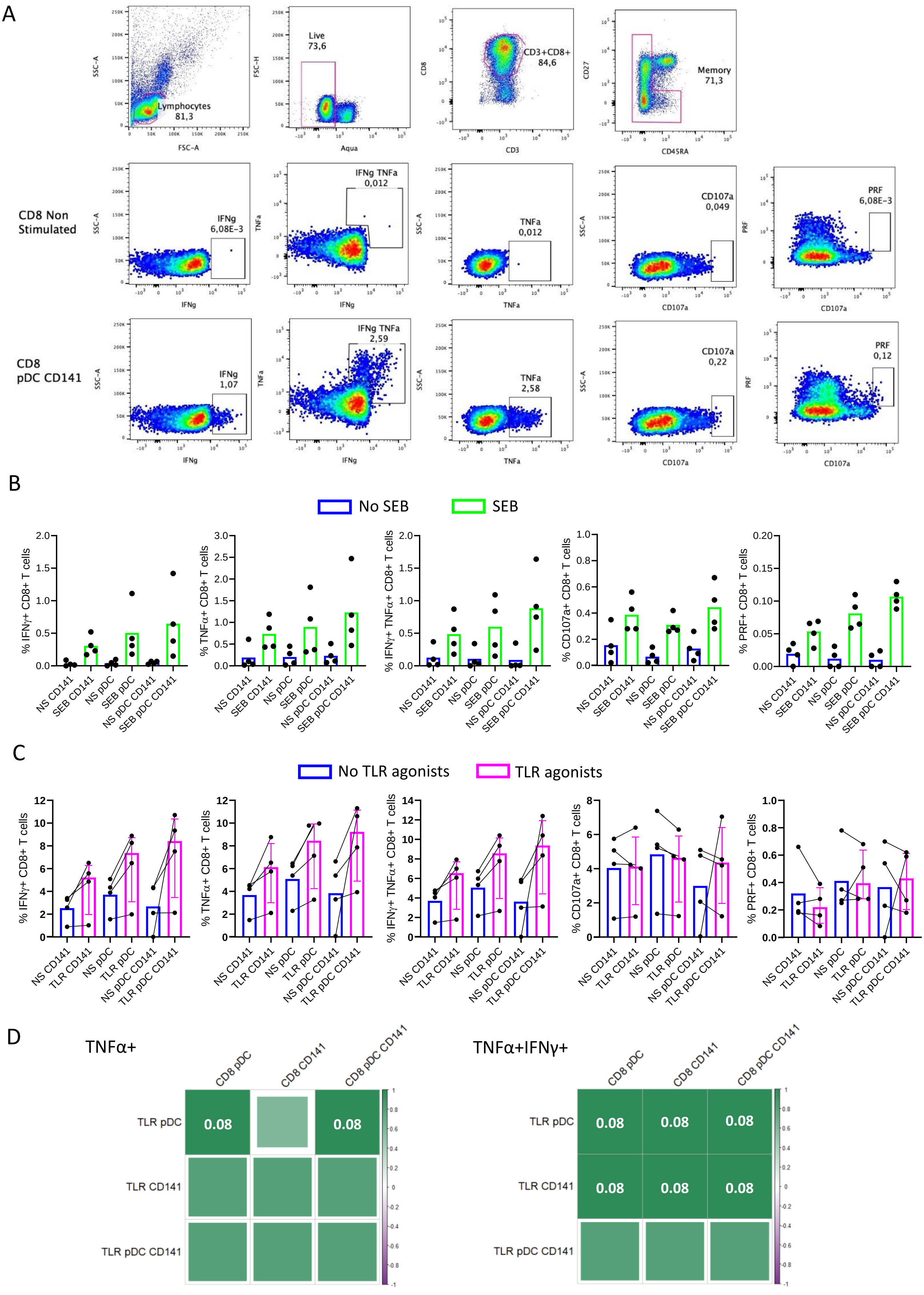

A
Lymphocytes 81,3
Live 73,6
CD3+CD8+ 84,6
Memory 71,3
CD8 Non Stimulated
CD8 pDC CD141
B
No SEB
SEB
NS CD141
SEB CD141
NS pDC
SEB pDC
NS pDC CD141
SEB pDC CD141
C
No TLR agonists
TLR agonists
NS CD141
TLR CD141
NS pDC
TLR pDC
NS pDC CD141
TLR pDC CD141
D
TNFα+
TNFα+IFNγ+
CD8 pDC
CD8 CD141
CD8 pDC CD141
TLR pDC
TLR CD141
TLR pDC CD141

Figure S4. CD8+ T cell response after DC – CD8+ T cell co-culture. (A) Pseudocolor dot plots showing the gating strategy for memory CD8+ T cell identification and functional markers expression after *in vitro* DC-CD8+ T cell co-culture. To define the gating of cytokines and cytotoxic markers, the non stimulated condition (without SEB or HIV peptide) was used. **(B)** Bar graphs showing the percentages of IFN-γ+, TNF-α+, IFN-γ+TNF-α+, CD107+ and perforin (PRF)+ CD8+ T cells after pre-stimulated DC – CD8+ T cell co-culture in the presence (green) or absence (blue) of enterotoxin type B (SEB) in HD. **(C)** Bar graphs showing the percentages of IFNγ+, TNFα+, IFNγ+TNFα+, CD107a+ and perforin (PRF)+ CD8+ T cells after the co-culture with non-prestimulated (blue) and TLR-stimulated (pink) DCs in HD. **(D)** Correlation matrixes representing the associations between TLR-dependent increase (TLR prestimulated – non prestimulated) of CD8+ T cell response with DC-mediated induction of CD8+ T cell response (CD8+DCs – only CD8), regarding TNFα+ (left) and TNFα+IFNγ+ (right) production. Green and purple colors represent positive and negative correlations, respectively. The intensity of the color and the size of the squares indicate the R coefficient. Wilcoxon and Spearman tests were used (n = 4). P values between 0.05 and 0.1 were considered as tendency and were showed as numbers.

Figure S5

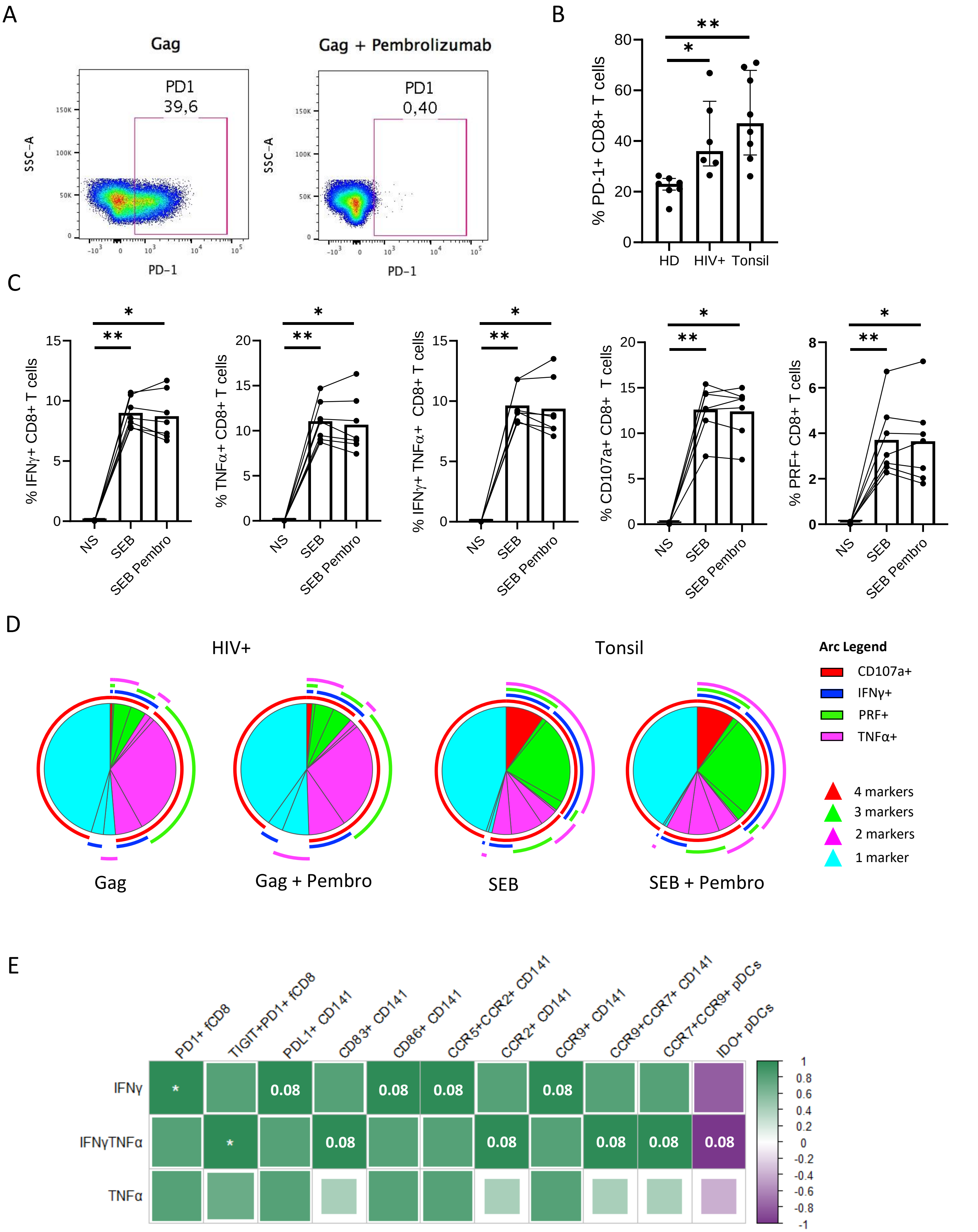

A
Gag
Gag + Pembrolizumab
PD1
39,6
PD1
0,40
SSC-A
PD-1
B
% PD-1+ CD8+ T cells
HD
HIV+
Tonsil
C
% IFNγ+ CD8+ T cells
% TNFα+ CD8+ T cells
% IFNγ+ TNFα+ CD8+ T cells
% CD107a+ CD8+ T cells
% PRF+ CD8+ T cells
NS
SEB
SEB Pembro
D
HIV+
Tonsil
Arc Legend
CD107a+
IFNγ+
PRF+
TNFα+
4 markers
3 markers
2 markers
1 marker
Gag
Gag + Pembro
SEB
SEB + Pembro
E
PD1+ fCD8
TIGIT+PD1+ fCD8
PDL1+ CD141
CD83+ CD141
CD86+ CD141
CCR5+CCR2+ CD141
CCR2+ CD141
CCR9+ CD141
CCR9+CCR7+ CD141
CCR7+CCR9+ pDCs
IDO+ pDCs
IFNγ
IFNγTNFα
TNFα

Figure S5. CD8+ T cell response after DC co-culture and PD-1 blockade with Pembrolizumab in HD. **(A)** Dot plot graphs showing representative data of PD-1 blockade efficiency in PWLH; the percentage of PD-1+ CD8+ T cells is shown. **(B)** Bar graphs representing the percentage of PD-1+ CD8+ T cells in blood of HD and PLWH and HD's tonsils. **(C)** Bar graphs showing the percentages of IFN-γ+, TNF-α+, IFN-γ+TNF-α+, CD107+ and perforin (PRF)+ CD8+ T cells in the presence of SEB in HD's blood after total cells stimulation with TLR agonists, in the presence and absence of pembrolizumab (Pembro). **(D)** Pie charts representing HIV-specific (left) and SEB-specific (right) CD8+ T cell polyfunctionality after DC - CD8+ T cell co-culture in PLWH and HD's tonsils, respectively, in the presence and absence of pembrolizumab. Each sector represents the proportion of CD8+ T cells expressing 4 (red), 3 (green), 2 (pink) and 1 (blue) function. Arcs represent the type of function (CD107a, IFN-γ, PRF, and TNF-α) expressed in each sector. **(E)** Correlation matrix representing the associations between the induction of CD8+ T cell cytokine production after PD-1 blockade (Gag+Pembrolizumab – only Gag) with *ex vivo* frequencies of specific subsets of fCD8, CD141 mDCs and pDCs in LN of PLWH. Green and purple colors represent positive and negative correlations, respectively. The intensity of the color and the size of the squares indicate the R coefficient. Kruskal–Wallis, Friedman, Permutation and Spearman tests were used (HD n=7, HIV+ n=6, tonsil n=8). *$P < 0.05$, **$P < 0.01$. P values between 0.05 and 0.1 were considered as tendency and were showed as numbers.

Supplementary Table 2

Fluorochrome-conjugated markers for *ex vivo* phenotyping

Marker	Fluorochrome	Clone	Company
DC panel			
Lin3	FITC		BD Biosciences
CD11c	BUV661	B-ly6	BD Biosciences
HLA-DR	PE-Cy5.5	TU36	Thermofisher
CD123	BV421	GH6	Biolegend
BDCA2	BV785	201A	Biolegend
CD1c	BV510	LI61	Biolegend
CD141	BV650	1A4	BD Biosciences
CD86	BUV737	2331	BD Biosciences
CD83	BUV395	HB15e	BD Biosciences
CD40	PE-Cy5	5C3	BD Biosciences
PD-L1	PE-Cy7	MIH1	BD Biosciences
CCR7	Ax700	150502	BD Biosciences
pDC panel			
BDCA2	BV785	201A	Biolegend
CD123	PE-Cy5	6H6	Biolegend
CD81	BV711	J5/81	BD Biosciences
CD5	BUV737	UCH72	BD Biosciences
CD2	PE-TexaRed	RPA-2.10	Thermofisher
CD103	BV711	BerACT8	BD Biosciences
CCR7	Ax700	150502	BD Biosciences
TLR9	PE	eB72-1665	BD Biosciences
Tim-3	BV421	F38-2E2	Biolegend
IDO	PE-Cy7	eyedio	eBioscience
T cell panel			
CD4	PE-Cy5.5	S3.5	Termofisher
CD8	BV650	RPA-T8	BD Biosciences
CD27	PE-Cy5	1A4CD27	Beckman Coulter
CD45RO	ECD	UCHL1	Beckman Coulter
PD-1	BV711	EH12.2H7	Biolegend
CCR7	AF700	G043H7	Biolegend
CXCR5	AF488	RF852	BD Biosciences

Figure S6

A

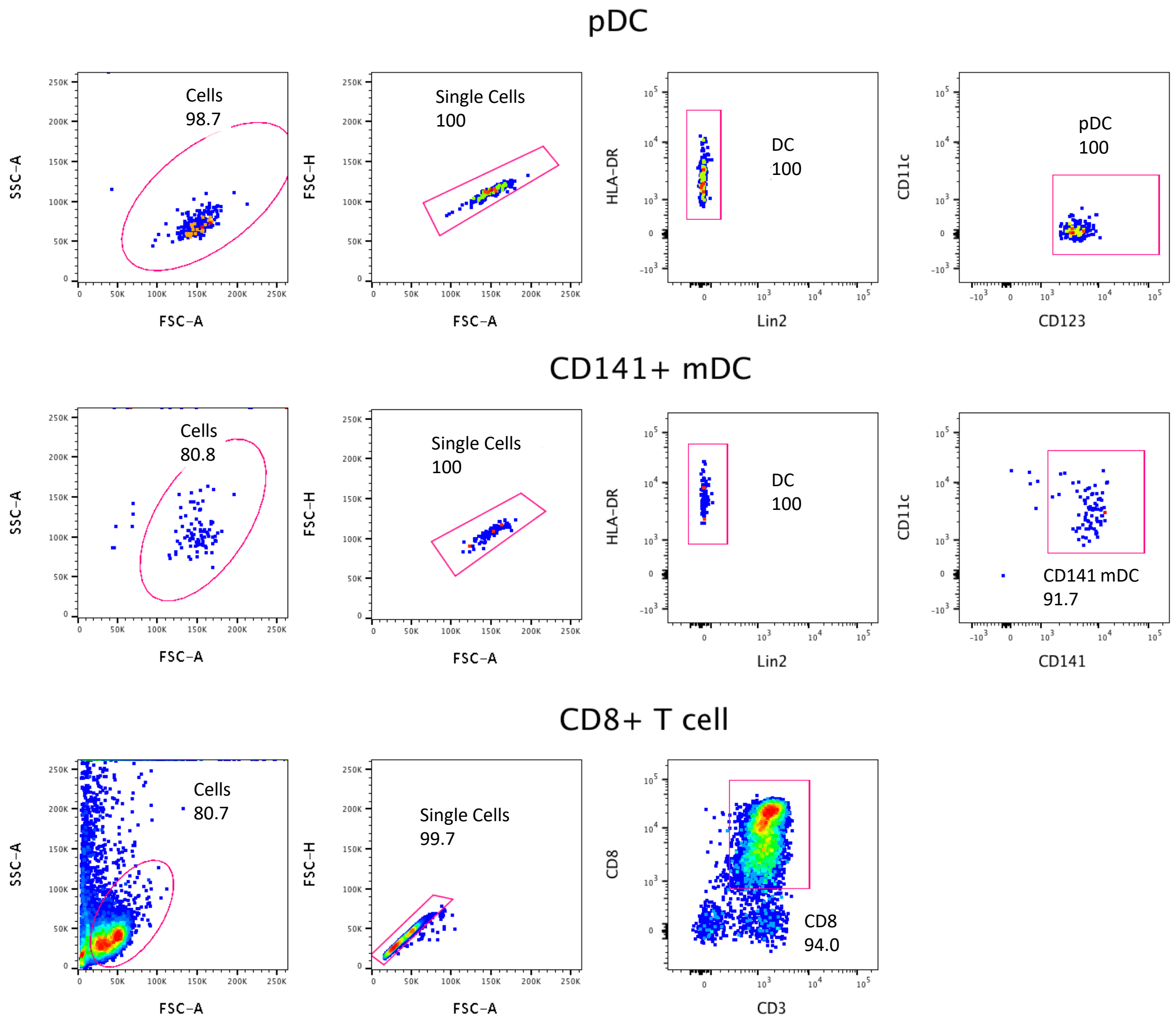
pDC
Cells
98.7
Single Cells
100
DC
100
pDC
100
SSC-A
FSC-H
HLA-DR
CD11c
FSC-A
Lin2
CD123
CD141+ mDC
Cells
80.8
Single Cells
100
DC
100
CD141 mDC
91.7
CD141
CD8+ T cell
Cells
80.7
Single Cells
99.7
CD8
94.0
CD8
CD3

B

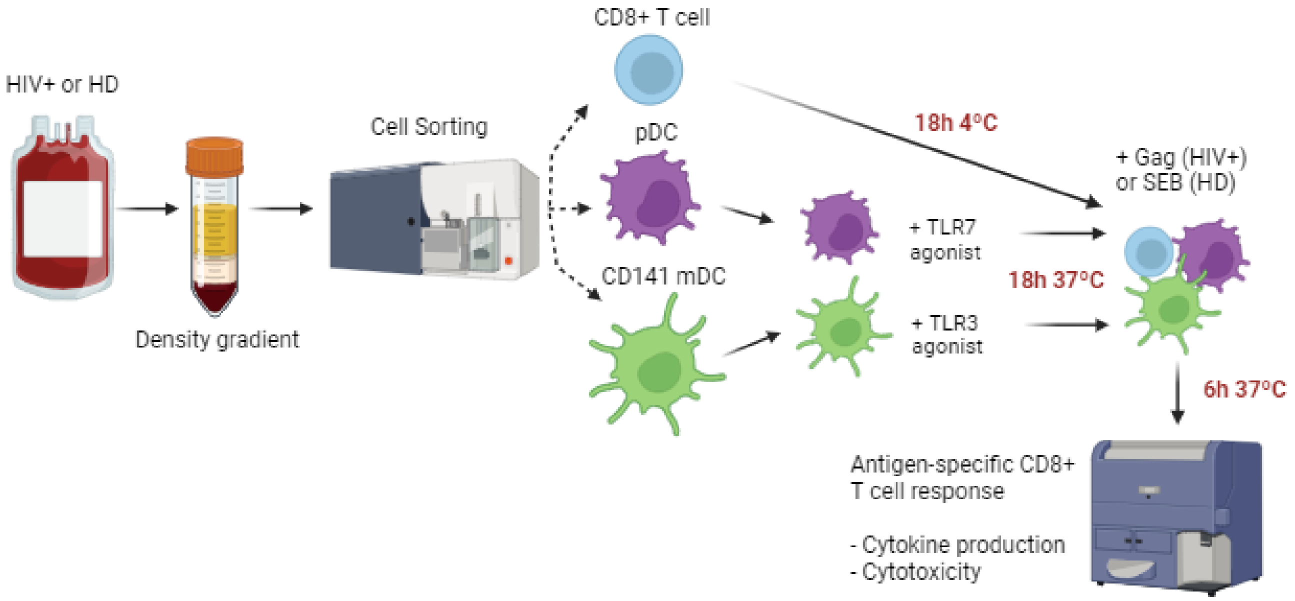
HIV+ or HD
Density gradient
Cell Sorting
CD8+ T cell
pDC
CD141 mDC
18h 4°C
+ TLR7
agonist
+ TLR3
agonist
+ Gag (HIV+)
or SEB (HD)
18h 37°C
6h 37°C
Antigen-specific CD8+
T cell response
- Cytokine production
- Cytotoxicity

Figure S6. Cell isolation and *in vitro* DC – CD8+ T cell co-culture. (A) Dot plot graphs showing a representative example of pDC, CD141+ mDC and CD8+ T cell isolation and purity. **(B)** Schematic representation of the followed protocol for *in vitro* cell stimulation, DC – CD8+ T cell co-culture and flow cytometry analysis (Created with BioRender.com).
